# Using the Social Ecological Model to identify barriers to accessing and using primary healthcare services by the rural Bangladeshi elderly: A systematic review

**DOI:** 10.1371/journal.pone.0358477

**Published:** 2026-09-18

**Authors:** Dipika Chandra, Sanjoy Kumar Chanda, Tunvir Ahamed Shohel, Bijoy Krishna Banik, Vinami Yulian

**Affiliations:** 1 Sociology Discipline, Social Science School, Khulna University, Khulna, Bangladesh; 2 Department of Sociology, Faculty of Social Science, University of Rajshahi, Rajshahi, Bangladesh; 3 School of Nursing, Faculty of Health Sciences, Universitas Muhammadiyah Surakarta, Surakarta, Indonesia; Hirosaki University School of Medicine & Hospital, JAPAN

## Abstract

**Background:**

Bangladesh's government is focusing on the United Nations Sustainable Development Goal 3 to improve primary healthcare (PHC) services, although rural elderly populations still lack access. The systematic review aimed at exploring and synthesizing the multilevel barriers to accessing and using PHC services by the rural Bangladeshi elderly based on the Social Ecological Model (SEM).

**Method:**

After completing registration in PROSPERO, electronic searches were conducted in March 2026 for a systematic literature search in the Scopus, PubMed, and ProQuest Sociological Abstracts databases, as well as in Google and Google Scholar. After a rigorous search, this review included 19 of 2099 articles, following the inclusion and exclusion criteria of the review. The quality of the articles was assessed using the Critical Appraisal Skills Programme and Mixed Methods Appraisal Tools. Barriers were identified using the thematic synthesis approach guided by the SEM.

**Results:**

The 19 included articles were synthesized into five themes: individual, family, organizational, social and community, and policy barriers. The review findings revealed that the key individual-level barriers included less physical capability, financial insolvency, and lack of PHC service knowledge. The family-level barriers included less connectivity with family and relatives, family influence over healthcare decisions, and gender disparity in families. The organizational-level barriers incorporated limited infrastructural facilities, unsatisfactory attitudes of healthcare providers (HPs), preference for gender-specific HPs, and treatment expenses. Dependence on traditional healers and lack of mobility were the social and community-level barriers. Finally, the policy-level barriers included rural elderly health insurance gaps.

**Conclusion:**

The review recommends PHC awareness campaigns, family support, simplification of PHC service-related facilities, social assistance, and health coverage programs to help rural Bangladeshi elderly access PHC services.

## Introduction

Although the United Nations (UN) Sustainable Development Goal 3 (SDG3) has a call to ensure health and well-being for all, the growing number of the elderly has been reported as a challenge to meet the healthcare requirements worldwide at present [1,2]. In Bangladesh, the emerging aging issue is associated with several challenges, such as lack of financial security, feelings of loneliness and exclusion, frequent sufferings of illness, and inadequate health facilities [3, 4]. The health vulnerability of the elderly is more acute than that of adolescents and young people [5]. As a result, the elderly suffer from a wide range of chronic diseases, such as diabetes, cancer, and heart attacks [2, 6]. In Bangladesh, healthcare services are provided through three levels, including primary, secondary, and tertiary [7]. The primary healthcare (PHC) service denotes the first stage of interaction between individuals and the organizational healthcare systems, which provides accessible, integrated, continuous, and individual-focused basic healthcare facilities [8]. The elderly generally prefer to seek easily available and accessible PHC services for illness treatment [9]. In rural Bangladesh, seeking healthcare services by the rural elderly is highly influenced by their socioeconomic condition, familial decisions, cultural issues, and the provision of institutional services [10, 11].

In the last few decades, although progress in many health indicators has been noticed, people, particularly the elderly, from the Low- and Middle-Income Countries (LMICs), still face substantial challenges such as lack of essential medicine facilities in healthcare centers [12, 13]. Additionally, studies showed that many of the rural elderly were unable to access and use PHC services due to insufficient legal frameworks, such as health allowances and insurance driven for elderly care and healthcare support [14]. On the contrary, several studies found that elderly people were reluctant to seek institutional healthcare services due to a lack of professionalism, knowledge, and skills of healthcare providers (HPs) [10, 15].

It is noteworthy that Bangladesh has set a target to achieve the SDG3 targets to promote healthy lives by 2030 [5, 16]. The Government of Bangladesh (GoB) has its health policy, which aims to deliver quality healthcare facilities to all its citizens, alongside special attention to children, adolescents, women, and disadvantaged rural people [17]. However, similar to other developing countries, the GoB lacks a comprehensive health policy and program for the rural elderly [5, 16]. Though the previous studies have shown that the health vulnerability of rural elderly remains a significant concern across the country [14, 18], the healthcare system of Bangladesh is inadequately prepared to address the growing healthcare needs of the elderly [19]. Research on the barriers to PHC services faced by the rural elderly in Bangladesh is scarce. However, the prior studies were limited to the rural elderly women, and very few studies were undertaken solely on socioeconomic and cultural impediments to avail PHC services of the elderly living in rural Bangladesh [14, 19–21]. Furthermore, under the healthcare framework of GoB, there are many health programs, such as maternal healthcare, child healthcare, and adolescent healthcare, but there is no specific program for elderly healthcare [22]. Although the Ministry of Social Welfare (MoSW) formulated the National Policy on Elder Persons 2013 to ensure social security for the elderly [23], no policy direction about the implementations of the policy was found due to the lack of substantial funding, investment, and human resources [24]. This systematic review provides evidence on the barriers to accessing PHC services among the elderly, highlights the necessity of improving healthcare services for the elderly to achieve SDG3, and informs evidence-based policies to improve accessibility of PHC services for the elderly, particularly in rural areas of Bangladesh. Consequently, this systematic review aimed at exploring and synthesizing the multilevel barriers to accessing and using PHC services by the rural Bangladeshi elderly.

### Guiding framework: The Social Ecological Model (SEM)

**Fig 1:**
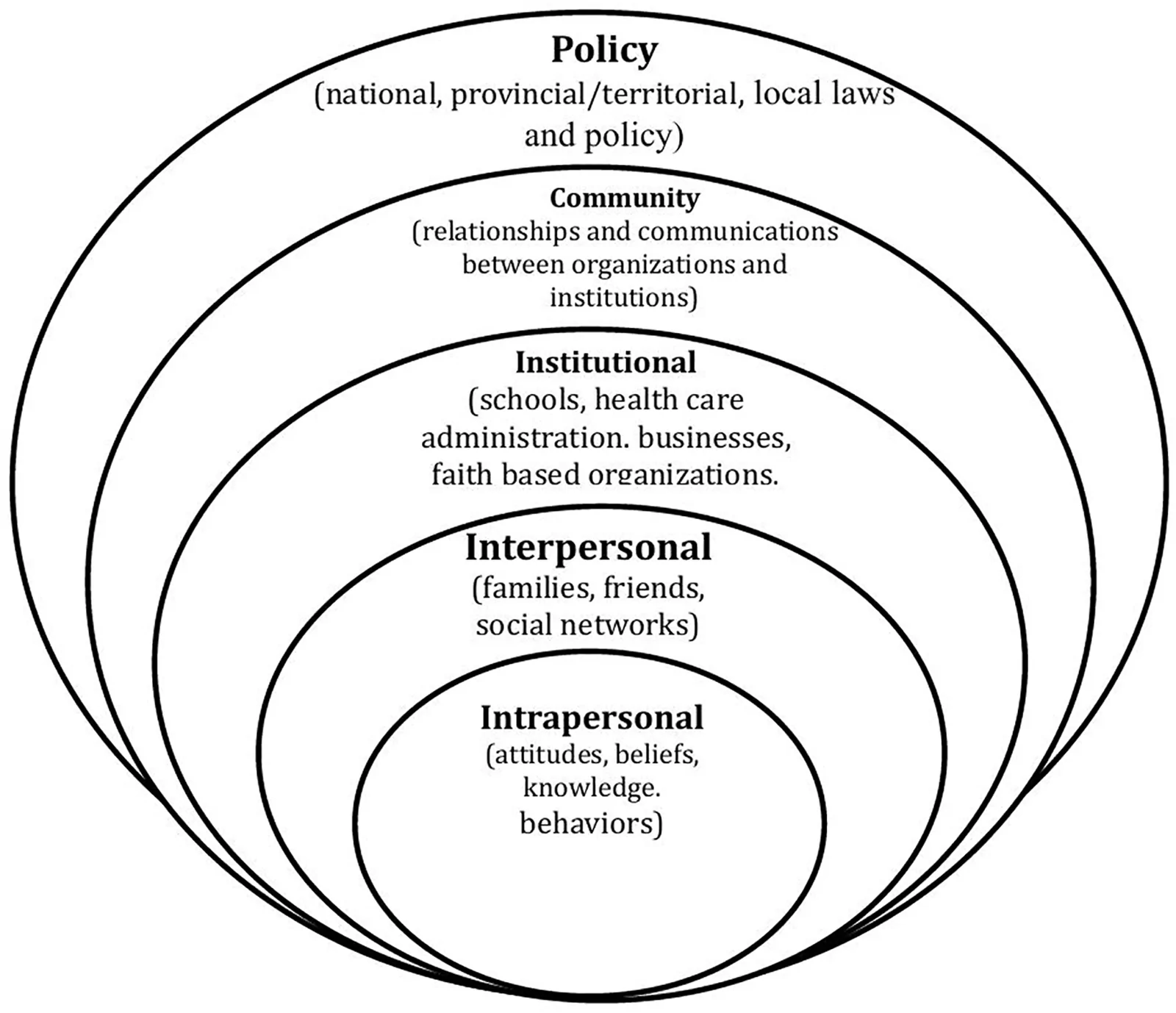
The Social Ecological Model. Adopted from Ma et al. (2017).

Several models, such as the Health Belief Model (HBM), Andersen’s Behavioral Model, and the SEM, are used in health research [25–28]. HBM gives more emphasis on an individual’s desired actions towards health and illness [29], and Andersen’s revised Behavioral Model predominantly focuses only on health outcomes of an individual but provides less understanding of psychological, social, and cultural mechanisms to utilize healthcare services [30, 31]. However, this paper used the SEM over other models, as this model provides multilevel understanding of barriers faced by the elderly in accessing PHC services, a feature that is absent in the other two models. Adopting the ideas from Bronfenbrenner’s Ecological Systems Theory (1977), McLeroy et al. (1988) first systematically applied SEM as a comprehensive framework to health promotion for understanding data from multiple perspectives (see Fig 1) [27, 32, 33]. This model conceptualizes the multidimensional determinants at the intrapersonal-level (e.g., age, sex, occupation, income, savings, etc.), the interpersonal-level (e.g., relationship with spouse, family members, and PHC service providers), the institutional-level (e.g., access and facilities in healthcare service centers), the social and community-level (e.g., sociocultural beliefs and community practices), and the policy-level (e.g., scarcity in health benefits coverage, healthcare policies and regulations, etc.) [27, 34]. This review adopted the SEM to provide a holistic understanding of barriers faced by the elderly in accessing and using PHC services and organize the findings identified from the studies included in this review.

## Materials and methods

### Study design

This systematic review was registered in the PROSPERO database (the International Prospective Register of Systematic Reviews; with the identification number CRD420251164335), the website of the National Institute for Health and Care Research (NIHR) [35] (see S1 File), and conducted following the Preferred Reporting Items for Systematic Reviews and Meta-Analyses (PRISMA) guidelines as a systematic framework for reporting reviews [36] (see S2 File). A systematic review was deemed the most suitable approach to comprehensively understand the barriers faced by the rural elderly in accessing PHC services, given the diverse nature of the literature, rather than merely summarizing findings in relation to a specific research question, as would be done in a narrative review [37].

### Eligibility criteria

This review included full-text, peer-reviewed studies that addressed barriers to accessing and using PHC services by the rural elderly (60 years and above) in Bangladesh. The timeline chosen for the selected studies was set from January 2000 to 2026 to identify the barriers to accessing healthcare facilities for the rural elderly in the 21st century and to avoid redundancy and overlapping of prior data. Additionally, the year 2000 signified the commencement of national and global actions for promoting PHC services, and studies limited to 2026 permitted this review to include the latest evidence regarding healthcare access for the elderly. Moreover, only scholarly studies were included in this review, as grey literature contained an absence of peer review, lacked standardized quality assessment, had incomplete or inconsistent research methods and results, increased the risk of bias, and limited accessibility to literature sources. Table 1 includes more detailed information about the eligibility criteria.

**Table 1:** Eligibility criteria for selection of studies.

Criteria	Inclusion criteria	Exclusion criteria
**Text**	Full-text peer-reviewed studies	Conference papers, abstracts, non-peer-reviewed studies, and newspaper articles
**Research method**	Qualitative, Quantitative, and Mixed Methods	Review
**Aim**	Studies focusing on access and use of PHC or healthcare services and barriers of PHC and healthcare services	Studies highlighting secondary and tertiary healthcare access and utilization
**Age**	60 years old and above	Below 60 years old
**Participants**	Elderly (both male and female)	Children, adolescents, and adult people
**Region**	Rural areas of Bangladesh	Developed countries, least developed countries, and only urban areas
**Limit of duration of publication**	Studies published from 2000 to 2026	Studies published before the year 2000

### Search strategies

Systematic and manual search strategies were applied to find out the studies included in this review. A Systematic search was undertaken with three electronic databases, including Scopus, PubMed, and ProQuest Sociological Abstracts, and a manual search with Google Scholar and Google on March 3, 2026 (see Table 2). The Population, Exposure, Outcome (PEO) Model was used to find out the relationship between exposure and health outcomes [38], and to develop search terms from the research question: What are the barriers elderly people face in accessing and using PHC services in rural areas of Bangladesh? Keeping in mind the research question, the PEO components identified in the study were: P: elderly (60 years and above); E: barriers; O: outcome (PHC access and utilization) The key search terms included in electronic databases, Google Scholar, and Google were: “healthcare”, “access to primary healthcare services”, “aging and barriers to healthcare facilities”, “limitations of primary healthcare services for the elderly”, and “healthcare impediments for older persons in rural Bangladesh”. The eligibility criteria for screening studies from Google Scholar and Google search were similar to the electronic search (see Table 1). Table 2 presents the details of the search terms in the electronic databases and the number of items included in each database.

**Table 2:** Applied search strategy and number of items found in the electronic database.

No. of search	Search term	No. of items
**Scopus**		
**#1**	Healthcare OR Primary healthcare AND Aged people OR Elderly people OR Older people OR Senior citizens OR People aged ≥ 60 and above AND Factors OR Barriers OR Determinants OR Indicators OR Challenges OR Impediments	**190,015**
**#2**	“Healthcare AND Aged People” OR “Healthcare AND elderly people” “Healthcare AND Older People” OR “Healthcare AND Senior Citizens” OR “Healthcare AND People aged 60 years and above”	**232,560**
**#3**	“Primary Healthcare AND Aged People” OR “Primary Healthcare AND elderly people” “Primary Healthcare AND Older People” OR “Primary Healthcare AND Senior Citizens” OR “Primary Healthcare AND People aged 60 years and above”	**210, 326**
**#4**	“Factors AND Primary Healthcare” OR “Barriers AND Primary Healthcare” OR “Factors AND Healthcare” OR “Barriers AND Healthcare”	**28,766**
**#5**	“Bangladesh” OR “Rural Bangladesh”	**64, 824**
**#6**	“Healthcare OR Primary healthcare” AND Aged people OR Elderly people OR Older people OR Senior citizens OR People aged ≥ 60 and above AND Factors OR Barriers OR Determinants OR Indicators AND Bangladesh	**9,951**
**#7**	“PUBYEAR, 2026” OR “LIMIT-TO (PUBYEAR, 2000)”	**17, 249**
**#8**	#1 AND #2 AND #3 AND #4 AND #5 AND #6 AND #7	**1,607**
**PubMed**		
**#1**	elderly OR elders OR rural elders OR older people AND healthcare OR primary healthcare* AND determinants OR challenges AND healthcare OR primary healthcare [All fields]	**670,833**
**#2**	elderly OR elders OR rural elders AND healthcare OR primary healthcare*	**625,269**
**#3**	aged people AND healthcare OR primary healthcare*	**599,648**
**#4**	60* years old* AND above OR 60 ≥ * years AND healthcare OR primary healthcare*	**141,005**
**#5**	primary healthcare services OR healthcare services OR primary care services or medical care OR primary medical care OR emergency medical care OR community healthcare* AND elderly*	**181,127**
**#6**	access to primary healthcare services OR healthcare services OR primary healthcare services* or medical care* OR primary medical care* OR emergency medical care* OR community healthcare* AND elderly OR rural elderly* OR rural elders*	**166,516**
**#7**	elderly OR rural elderly OR rural elders AND hindrances OR impediments OR influencing factors/ determinants OR socioeconomic* determinants/ factors OR decision making* determinants/ factors or cultural* determinants/ factors OR challenges in healthcare services	**26,344**
**#8**	utilization of primary healthcare services OR use of healthcare services OR use of primary healthcare services OR medical care OR primary medical care OR emergency medical care OR community health care OR practice of healthcare services AND elderly OR elderly* OR rural elders*	**46, 518**
**#9**	((“elderly” AND access to healthcare) AND hindrances) OR (“aged” people AND access to primary health) AND hindrances OR impediments OR barriers OR challenges))	**68,398**
**#10**	(((“elderly” AND healthcare) AND determinants [All Fields])) AND Bangladesh))) AND rural areas)))) OR (((“aged” people AND healthcare) AND determinants [All Fields])) AND Bangladesh))) AND rural areas)))	**5,220**
**#11**	(((“60 ≥ years” * AND healthcare) AND Barriers [All Fields]) AND Bangladesh)) AND rural areas))) OR ((“60 AND above or years old” * AND healthcare) AND barriers [All Fields])) AND Bangladesh)) AND rural areas)))	**1, 842**
**#12**	((((“elderly” AND access) AND healthcare)) OR ((“elderly” AND use) AND primary healthcare)) OR ((“aged” people AND impediments [All Fields])) AND healthcare)) OR ((“aged” people AND hindrances [All Fields])) AND primary healthcare)) OR ((“older” people AND challenges [All Fields])) AND healthcare)) AND rural areas))) AND Bangladesh*))))	**476**
**ProQuest Sociological Abstract**		
**S1**	Healthcare OR Primary healthcare AND Aged people OR Elderly people OR Older people OR Senior citizens OR People aged ≥ 60 and above AND Factors OR Barriers OR Determinants OR Indicators OR Challenges OR Impediments	**6,20,365**
**S2**	Elderly OR Aged people OR Older people OR Senior citizens OR (60 years and above)	**53, 426**
**S3**	Healthcare OR Healthcare services OR Primary Healthcare OR Primary healthcare services OR Medical Care	**12, 342**
**S4**	Barriers OR (Socioeconomic impediments) OR (Institutional impediments) OR (Access, healthcare AND factors) OR (Use, healthcare AND determinants) OR (Access, primary healthcare AND challenges) OR (Utilization, primary healthcare AND challenges)	**27, 540**
**S5**	Add Bangladesh	**2, 621**
**S6**	Limit to year 2000–2026	**1, 200**
**S7**	S1 AND S2 AND S3 AND S4 AND S5 AND S6.	**16**

### Selection of the studies

**Fig 2:**
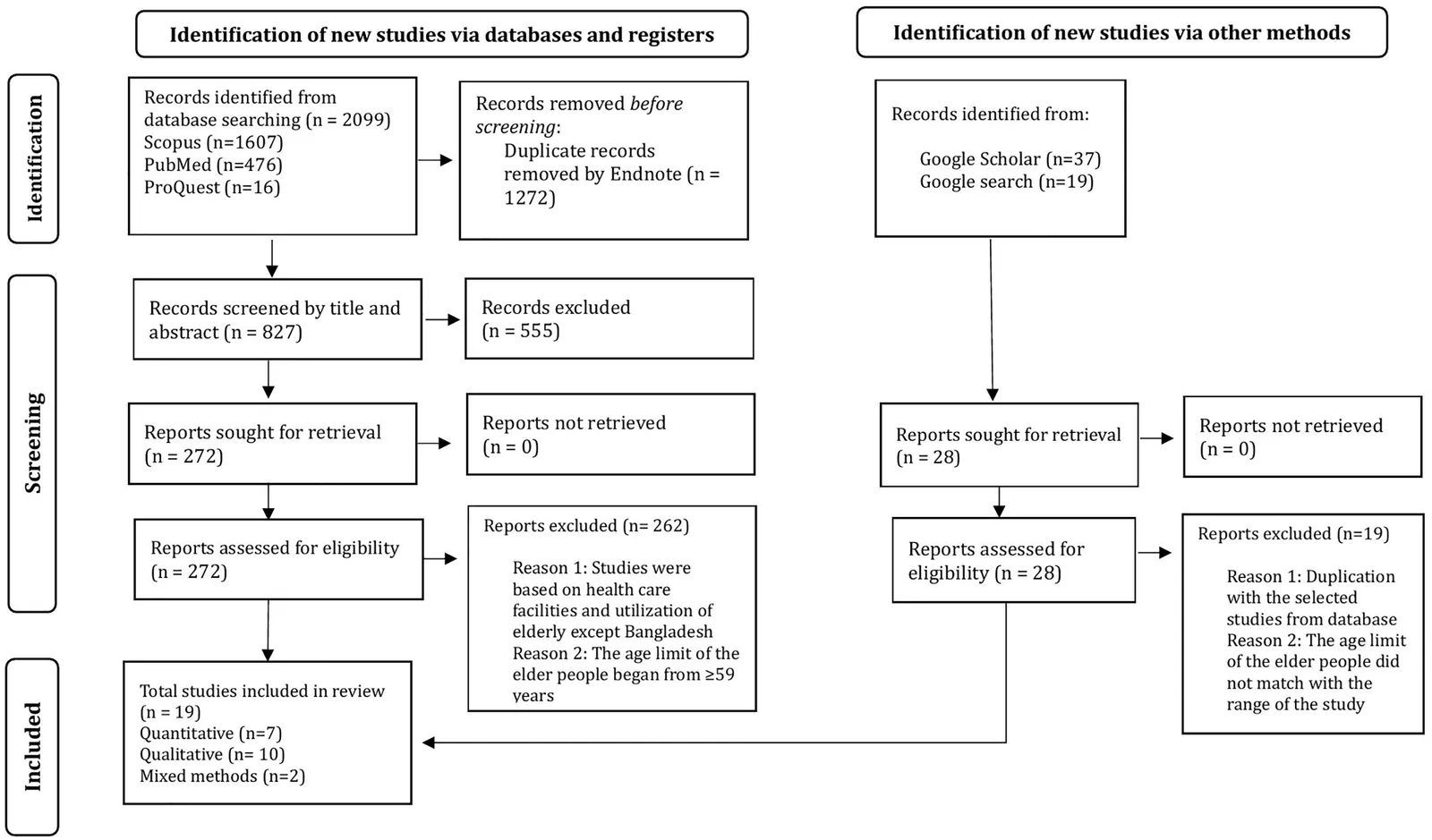
PRISMA flowchart for searches of the database and records. Adopted from Page et al. (2020).

A total of 2099 studies were initially identified through database searching, of which 1272 were removed because of duplication. The process of removing duplication was completed using EndNote software (v 21) and an intensive manual process by checking every study, while the rest (827 studies) were taken for screening. The first and second authors of this study (DC and SC) independently screened the titles, abstracts, and objectives of the retrieved publications based on the eligibility criteria outlined above. Additionally, the studies found similar to the eligibility criteria of this study, were moved at full-text review screening step. Among 827 studies, 555 were excluded as those studies did not match the current research topic. Finally, the full text of 272 studies was gone into detail and based on the exclusion criteria (see Table 1), the remaining 262 studies were again excluded. The full text of the eligible studies was available from the open-access journals, and if any study was not available, the lead author of that study was requested through email for access. Furthermore, from additional searches with Google and Google Scholar, 9 studies were included from 28 assessed studies. Finally, a total of 19 studies were considered eligible for review based on the inclusion criteria. There was no disagreement between the two investigators about the final decision to select 19 studies. The PRISMA flowchart was adopted to outline the details of the literature search and selection procedure [36] (see Fig 2).

### Data extraction

Data were extracted independently by DC and SC using a standardized Excel checklist form agreed upon by the researchers of this study. Inter-rater reliability was assessed using Cohen’s kappa (κ = 0.75, 95% CI 0.71–0.82), indicating substantial agreement [39]. Each eligible study was checked by this standardized checklist, including (i) authors name, publication year, and country; (ii) age and sex of the participants; (iii) total sample size; (iv) study design; (v) study timeframe; and (vi) findings of the study (morbidity pattern of the participants, healthcare seeking behavior of the participants, and barriers related to accessing healthcare services or PHC services by the participants) for extracting data and the discrepancies were resolved with consensus by TAS.

### Assessing the quality of the included studies

The quality of the selected studies was assessed before including them in this review study. The quality of quantitative cross-sectional studies was assessed by the checklist of Critical Appraisal Skills Programme (CASP), adopted from Crombie [40]. The CASP included 12 questions with three possible answers: ‘Yes’, ‘Can’t tell’, and ‘No’ to assess research design, participants, data collection tool, satisfaction of response, measurement of reliability, and statistical significance (see Table 3).

**Table 3:** Appraisal tools for studies included in this literature review.

Appraisal tools for quantitative cross-sectional studies (Survey) (Y= Yes, C= Can’t tell, N=No)																		
Author, Year, Country	1. Did the study address a clearly focused question / issue?	2. Is the research method (study design) appropriate for answering the research question?	3. Is the method of selection of the subjects (employees, teams, divisions, organizations) clearly described?	4. Could the way the sample was obtained introduce (selection) bias?	5. Was the sample of subjects’ representative with regard to the population to which the findings will be referred?	6. Was the sample size based on pre-study considerations of statistical power?	7. Was a satisfactory response rate achieved?	8. Are the measurements (questionnaires) likely to be valid and reliable?	9. Was the statistical significance assessed?	10. Are confidence intervals given for the main results?	11. Could there be confounding factors that haven’t been accounted for?	12. Can the results be applied to your organization?	Risk-of-bias					
1. Sarker, 2025, Bangladesh	Y	Y	Y	N	Y	Y	Y	Y	Y	Y	Y	Y	Low					
2. Akter et.al. 2023, Bangladesh	Y	Y	Y	N	Y	Y	Y	Y	Y	Y	N	Y	Some concerns					
3. Islam et.al., 2022, Bangladesh	Y	Y	Y	N	Y	Y	Y	Y	Y	Y	N	Y	Some concerns					
4. Rummi et.al., 2022	Y	Y	Y	N	Y	Y	Y	Y	Y	Y	N	Y	some concerns					
5. Ferdaus et.al., 2020, Bangladesh	Y	Y	Y	N	Y	Y	Y	Y	Y	Y	N	Y	Some concerns					
6. Hossian et.al., 2019, Bangladesh	Y	Y	Y	N	Y	Y	Y	Y	Y	Y	N	Y	Some concerns					
7. Jabeen et, al., 2015, Bangladesh	Y	Y	Y	N	Y	Y	Y	Y	Y	Y	N	Y	Some concerns					
**Quality appraisal tools for qualitative studies (Y= Yes, C= Can’t tell, N=No)**																		
Author, Year, Country	1. Was there a clear statement of the aims of the research?	2. Is a qualitative methodology appropriate?	3. Was the research design appropriate to address the aims of the research?	4. Was the recruitment strategy appropriate to the aims of the research?	5. Was the data collected in a way that addressed the research issue?	6. Has the relationship between researchers and participants been adequately considered?	7. Have ethical issues been taken into consideration?	8. Was the data analysis sufficiently rigorous?	9. Is there a clear statement of findings?	10. How valuable is the research?	Risk-of-bias							
8. Sarker et.al. 2023, Bangladesh	Y	Y	Y	Y	Y	Y	Y	Y	Y	High	Low							
9. Hamiduzzaman et.al. 2023, Bangladesh	Y	Y	Y	Y	Y	Y	Y	Y	Y	High	Low							
10. Hamiduzzaman et.al., 2022b, Bangladesh	Y	Y	Y	Y	Y	Y	Y	Y	Y	High	Low							
11. Hamiduzzaman et.al., 2021a, Bangladesh	Y	Y	Y	C (The sampling method and sample size are not clearly mentioned.)	N	C (There is no rapport-building statement in the study.)	Y	Y	Y	High	7 (70.0) High							
12. Hamiduzzaman et.al., 2021b, Bangladesh	Y	Y	Y	Y	Y	Y	Y	Y	Y	High	Low							
13. Hossen & Westhues, 2012, Bangladesh	Y	Y	Y	Y	N	Y	Y	Y	Y	High	Some concerns							
14. Hossen & Westhues, 2011, Bangladesh	Y	Y	Y	Y	Y	Y	Y	Y	Y	High	Low							
15. Hossen & Westhues, 2010, Bangladesh	Y	Y	Y	Y	Y	Y	Y	Y	Y	High	Low							
16. Biswas, 2007, Bangladesh	Y	Y	Y	C (The sample groups were not clearly specified)	Y	Y	C (No ethical statement was reported)	Y	N (The triangulation of findings between FGDs and IDIs was not clearly reported)	High	High							
17. Biswas et.al, 2006, Bangladesh	Y	Y	Y	Y	Y	Y	C (No ethical statement was reported)	N	N (The study did not clearly present findings for elderly and their caregivers separately )	High	High							
**Quality appraisal tools for mixed methods studies (Y= Yes, C= Can’t tell, N=No)**																		
**Author, Year, Country**	**Screening questions (for all types)**		**1. Qualitative studies**					**2. Quantitative studies**					**3. Mixed methods study**					**Risk-of-bias**
	S.1 Are there clear research questions?	S.2 Do the collected data allow to address the research questions?	1.1 Is the qualitative approach appropriate to answer the research question?	1.2 Are the qualitative data collection methods adequate to address the research question?	1.3 Are the findings adequately derived from the data?	1.4 Is the interpretation of results sufficiently substantiated by data?	Is there coherence between qualitative data sources, collection, analysis and interpretation?	2.1 Is the sampling strategy relevant to address the research question?	2.2 Is the sample representative of the target population?	2.3 Are the measurements appropriate?	2.4 Is the risk of nonresponse bias low?	2.5 Is the statistical analysis appropriate to answer the research question?	3.1 Is there an adequate rationale for using a mixed methods design to address the research question?	3.2 Are the different components of the study effectively integrated to answer the research question?	3.3 Are the outputs of the integration of qualitative and quantitative components adequately interpreted?	3.4 Are divergences and inconsistencies between quantitative and qualitative results adequately addressed?	3.5 Do the different components of the study adhere to the quality criteria of each tradition of the methods involved?	
18. Hamiduzzaman 2020, Bangladesh	Y	Y	Y	Y	Y	Y	Y	Y	Y	Y	Y	Y	Y	Y	Y	Y	Y	Low
19. Abdullah et.al., 2018, Bangladesh	Y	Y	Y	Y	Y	Y	Y	Y	Y	Y	Y	Y	Y	Y	Y	Y	Y	Low

The checklist of CASP for qualitative studies (see Table 3) was also adopted to address the quality of the selected qualitative studies [41, 42]. The checklist contained 10 questions with possible answers: ‘Yes’, ‘Can’t tell’, and ‘No’ to examine the aim, method, participants, rigor and findings of the qualitative research.

For Mixed Methods studies (MMs), 17 questions of the MMs Appraisal Tool (MMAT), version 2018 [43], were used for critical appraisal of the studies (see Table 3). Among the seventeen items, the first two were screening questions, and the rest 15 were related to qualitative, quantitative, and MMs studies with possible answers: ‘Yes’, ‘Can’t tell’, and ‘No’.

To assess the quality of the studies included in the systematic review, the three Cochrane risk‑of‑bias levels—low, some concerns, and high—were adopted [44]. The studies were evaluated based on (1) signaling questions with possible responses: Yes, Can’t tell, and No; and (2) descriptive responses for open‑ended questions. A low risk of bias was assigned when all responses were met; some concerns when one or two responses were unmet; and high risk of bias when three or more responses were unmet.

DC and SC independently assessed the quality of the included studies, and discrepancies were resolved through consensus meetings, during which DC and SC justified their ratings outlined in the appraisal tools. Inter-rater reliability was assessed using Cohen’s kappa (κ = 0.75, 95% CI 0.71–0.82), indicating substantial agreement [39].In case of disagreement, other co-authors were consulted to negotiate the rankings and make the final decision.

### Data synthesis

In recent years, several methods, such as meta-analysis, meta-synthesis, and thematic synthesis, have emerged to synthesize data from existing literature related to health research [45, 46]. Meta-analysis focuses on objective idealism (quantitative data synthesis), meta-synthesis deals with individual studies under review (qualitative data synthesis), and thematic synthesis accounts for constant comparison methods (multiple approaches to the study) [46, 47]. However, the thematic synthesis approach is widely preferred to arrange the free codes of findings into descriptive themes and later interpreted to analytical themes [46, 47]. Therefore, as the review included multiple natures of studies, such as qualitative, quantitative, and MMs, the thematic synthesis approach was found suitable for this review. The three phases of the data analysis process were used in this systematic review with thematic synthesis: coding text, developing descriptive themes, and generating analytical themes [45]. The codes, sub-themes and final themes were mostly organized deductively following the hierarchical order of SEM to identify the existing barriers to accessing and using PHC services by the rural elderly from the perspective of Bangladesh (see S1 Table). The line-by-line coding approach [48] was followed to identify code based on the SEM framework. Codes were developed independently by two authors (DC and SC). For the development of coding, we followed the Cohen’s kappa approach (κ = 0.75, 95% CI 0.71–0.82), representing significant consensus [39]. The characteristics of included studies were summarized and tabulated following the guidelines of Ma et al. (2017) [27]. The characteristics and significant findings comprised: (i) author(s), year of publication, and country where the study was conducted; (ii) aim of the study; (iii) study design; (iv) sampling method and participants; and (v) key findings: barriers to accessing and using PHC services by the rural elderly (see Table 4).

**Table 4:** Study characteristics and outcome summary of the included studies.

Author, Year, Country	Aim	Study design	Sampling Method & Participants	Key findings: Barriers to access and use PHC services by rural elders
1. Sarker, 2025, Bangladesh	To access factors influencing healthcare utilization among the rural elderly in Bangladesh	Quantitative (Cross-sectional survey)	Simple random sampling Elderly, aged 60 and above N = 585	• Lack of knowledge about illness, lack of education, marital status of the elderly, living arrangement, and financial condition significantly influenced healthcare utilization of the elderly
2. Akter et.al. 2023, Bangladesh	To explore the morbidity patterns and determinants of healthcare seeking behavior, among the elderly women in rural areas	Quantitative (Exploratory cross sectional)	Simple random sampling Women aged 60 and above years N = 233	• Lack of knowledge about healthcare facilities due to ignorance, reliance on family members on monetary issues and having no financial assistance for healthcare were major barriers to utilize healthcare facilities
3. Rummi et.al. 2022, Bangladesh	To illustrate sociodemographic status and evaluate the care seeking behavior of the rural elderly	Quantitative (Cross-sectional survey)	Purposive sampling Elderly of above 60 years N = 427	• Knowledge gap, poor socioeconomic status, non-cooperation of healthcare service providers, treatment cost were the notable barriers
4. Islam et.al., 2022, Bangladesh	To find out the socioeconomic and demographic characteristics of elderly, factors influencing health status of them and to describe the future situation of the elderly	Quantitative (Survey)	Multistage sampling Elderly (60 years and above) N = 316	• Financial dependency resisted the older respondents to avail proper healthcare amenities
5. Ferdaus et.al., 2020, Bangladesh	To find out risk factors associated with access to healthcare services by the elderly of rural areas	Quantitative (Cross- sectional survey)	Purposive sampling Elderly, aged 60 and above N = 50	• Lack of knowledge regarding illness treatment and illiteracy significantly hindered the healthcare access of the rural elderly
6. Hossian et.al., 2019, Bangladesh	To measure self‑reported ailments, patterns of healthcare seeking behavior as well as affordability for healthcare facilities, and to point out the implication for PHC service delivery in rural areas	Quantitative (Cross-sectional survey)	Multistage sampling Rural elderly N = 362	• Female respondents were reluctant to seek healthcare services for their aliment • A large portion of respondents visited unqualified healthcare providers • The elderly were dependent on traditional support system for healthcare expenditure • Village doctors and untrained healthcare services providers were preferred most by the respondents • Lack of institutional care facilities
7. Jabeen et, al., 2015, Bangladesh	To find it the ailment pattern and healthcare seeking patten of the rural elderly	Quantitative (Cross sectional survey)	Purposive sampling Elderly (60 years and above) N = 249	• Lack of availability of medicine, high cost of medicine, distance of the PHC centre and unsatisfactory behavior of the healthcare provider
8. Sarker et.al. 2023, Bangladesh	To gain insights on experiences of healthcare seeking along with the ailment expenditure, and to find out the accessibility and coping mechanisms to improve the quality of life of the elderly	Qualitative (IDIs)	Purposive sampling Elderly (aged 60 years and more) N = 27	• Absence of sufficient seats for patients as well as irregular checkups from doctors, and poor hygiene management of upazila health complex. • High treatment and medical cost • Financial dependency on others for treatment
9. Hamiduzzaman et.al. 2023, Bangladesh	To report the determinants of healthcare access by rural elderly women	Qualitative (IDIs and KIIs)	Purposive sampling Elderly women (aged 60 years and above) Older women (N = 25), and healthcare professionals (N = 11)	• Lack of awareness of illness, mistrust toward medical treatment, self-treatment, and religious values and norms at individual level, isolation in family and communication with clinicians at family level, community perception of aging, neighboring and lack of community organizations at community level, and care affordability, social safety-net coverage and lack of national health policy at policy level hindered the elderly women’s access to healthcare services in rural areas.
10. Hamiduzzaman et.al., 2022b, Bangladesh	To explore the system determinants that impact on rural elderly women’s use of healthcare	Qualitative (KIIs and IDIs)	Not clearly reported Healthcare staff and Elderly women (60 years and above years of age) KIIs (N = 11) IDIs (N = 25)	• Insufficient legal framework for age care, inadequate healthcare support, special care for children and pregnant women and lack of age specific professional knowledge of healthcare providers were revealed as major barriers
11. Hamiduzzaman et.al., 2021a, Bangladesh	To analyze the social as well as structural factors regarding PHC access and use of marginalized elderly women	Qualitative (Critical Social Framework (CSF))	Not clearly reported Elderly women (60 and above years old) N = Not reported	• Health policies and systems in Bangladesh were not framed to meet primary care needs of rural elderly women • Male dominated health practices, power practices of health professionals, lack of proper communication with care providers and limited decision-making capacity of the elderly women
12. Hamiduzzaman et.al., 2021b, Bangladesh	To focus on the social and individual factors of PHC access and use of the elderly women	Qualitative (Critical Social Framework (CSF))	Comprehensive sampling method Elderly women (aged 60 years and above) N = 25	• Long waiting time for health checkup, lack of well-equipped and costly medical tests, scarcity of doctors, especially female doctors • Lack of knowledge of the respondents regarding medical care and financial dependency on family members for ailment treatment
13. Hossen & Westhues, 2012, Bangladesh	To gain insights on the use of medical care among the elderly women living in rural areas	Qualitative (Feminist, phenomenological approach)	Not clearly reported Elderly women (60–75 years of age) N = 17	• Culturally sensitive practices • Poor infrastructural condition, especially in monsoon • Lack of healthcare service providers
14. Hossen & Westhues, 2011, Bangladesh	To find out the health status and healthcare seeking behavior, and to identify the barriers to utilization of healthcare services by the elderly women	Qualitative (Feminist, phenomenological approach)	Random sampling (Three stage process) Elderly women (60 ≥ years of age) N = 17	• Perceived discrimination based on age, class, and gender; structural aspects of the healthcare delivery system; and quality of care
15. Hossen & Westhues, 2010, Bangladesh	To explore the restrictions of the elderly women to access healthcare services	Qualitative (Feminist, phenomenological approach)	Random sampling (Three stage process) Elderly women (Aged 60 and above) N = 17	• Lack of social mobility, gender disparity, religious restrictions like *Purdah*, social and cultural stigma associated with illness, male centered decision-making process and financial limitations were the main barriers for treatment seeking of the elderly women
16. Biswas et.al, 2007, Bangladesh	To assess the elements of patient satisfaction that affect and influence healthcare use by the elderly in rural Bangladesh	Qualitative (FGD and IDI)	Non-random strategy (purposive sampling and opportunistic sampling) Elderly individuals aged 60 years and above FGD (N = 6) IDI (N = 30)	• Scarcity of human resources in health sector • Lack of health funding and social protection • Non-availability of geriatric care provider
17. Biswas et.al, 2006, Bangladesh	Focused on coping strategies, illness of the elderly and the determining factors of their healthcare seeking behavior	Qualitative (FGD and IDI)	Non-random sampling (stratified purposive sampling and opportunistic sampling) Elderly above 60 years of age and their caregivers N = 5 FGD (elderly), 4 FGD (caregivers) N = 30 interviews (elderly)	• Financial obstacle, expensive medical treatments, non-cooperation of medical care providers
18. Hamiduzzaman 2020, Bangladesh	To identify the health effects of seasonal variations on the rural elderly women, and to explore the determinants associated with the elderly women’s use of local healthcare	Mixed methods (Survey and KII)	Representative Rural elderly women (60 years old or over) Survey (N = 65) KII (N = 11)	• Scarcity of doctors, nurses and health assistants, limited allocation of medical equipment, waiting time, the lack of gerontology knowledge by the staff and financial inability to pay for treatment
19. Abdullah et.al., 2018, Bangladesh	To evaluate the accessibility to healthcare services for the elderly	Mixed methods (Survey, FGD and KII)	Random Elderly between 60 and 90 years of age Survey (N = 93) FGD (N = 4) KII (N = 4)	• Newly recruited young doctors generally provided the services at upazila hospital • The number of specialized medical practitioners was low and unavailable • In upazila hospital there is no geriatrician • Limited diagnostic facilities at upazila level health centres

## Results

### Characteristics of the selected studies

A summary of the characteristics of the studies included in this review is provided in Table 4. The 19 studies included in this review were published between 2006 and 2025, with the majority (n = 15; 78.95%) occurring between 2015 and 2025. Notably, no relevant study was found for the period 2000–2005. Of the 19 studies, ten studies used qualitative research design, seven quantitative, and two MMs. In quantitative research, data collection involved survey methods, while qualitative studies were undertaken using Focus Groups (FGs), In-Depth Interviews (IDIs), and Key Informant Interviews (KIIs). In the case of MMs research, quantitative data were first collected using the survey method, followed by qualitative data collection using FGs and KIIs. Out of 19 studies, nine had a low risk of bias, seven had some concerns, and three had a high risk of bias. Studies assigned a high risk of bias did not clearly report their sampling procedures, rapport‑building strategies, ethical clearance, or triangulation processes [11, 18, 49].

### Description of themes

Five key themes with several subthemes, representing the barriers to accessing and using PHC services by rural Bangladeshi elderly, were identified. The key themes included: (i) individual-level barriers; (ii) family-level barriers; (iii) organizational-level barriers; (iv) social and community-level barriers; and (v) policy-level barriers to accessing and using PHC services by the elderly people in rural Bangladesh (see S1 Table).

#### Theme 1: Individual-level barriers to accessing and using PHC services by rural elderly.

The theme of individual-level barriers to accessing and using PHC services included three subthemes: (i) demographic barriers, (ii) financial insolvency, and (iii) knowledge gap regarding PHC services. Additionally, this theme contained 15 studies, including six quantitative [50–55]; seven qualitative [11, 18, 56–60], and two MMs [22, 61].

(i) **Demographic barriers**

Two qualitative studies [57, 59] and two quantitative studies [51, 52] revealed that aging leads to a decline in physical ability in elderly people, which hinders them from accessing PHC services. For example, the people who belonged to the age of 80 years and above were more incapable of traveling than the elderly who were below 80 years of age [55, 57]. Additionally, three qualitative studies [18, 51, 56] reported not having a spouse, living alone, and illiteracy were the predominant barriers to seeking treatment for the rural elderly. It had been noticed that the elderly who were married or living with children used to avail PHC services two times more than the elderly who did not have a spouse or were living alone. This review also revealed that, being female, the elderly women were less likely to seek PHC services – [58, 61].

(ii) **Financial insolvency**

Five quantitative studies [50–53, 55] and an MMs study [61] focused on the fact that the financial insolvency of the rural elderly leads to a lack of making independent healthcare decisions, which prevents them from accessing PHC services. The review revealed that most of the elderly in rural areas become financially inactive with the growth of age and the severity of illness [52]. Consequently, they cannot afford the cost of the treatment by themselves and become reluctant to seek quality treatment from PHC centers [55, 61].

(iii) **Knowledge gap regarding PHC services**

This subtheme included two quantitative studies [54, 55], three qualitative studies [57, 58, 60], and one MMs study [22]. The review found that a substantial portion of the rural elderly were not aware of the places to seek PHC services, and the roles and functions of PHC service providers [22, 54, 55, 57, 58, 60]. For instance, the type of services provided in rural PHC centers, such as community clinic (CC), union sub-centers (USC), rural sub-center (RSC), upazila health complex (UHC), were not clearly known to the rural elderly women [57].

#### Theme 2: Family-level barriers to accessing and using PHC services by the rural elderly.

This theme consisted of three subthemes: (i) lack of familial support in seeking PHC services, (ii) influence of family members on making healthcare decisions, and (iii) family restrictions on receiving outside healthcare services for the elderly women. This theme included five qualitative [18, 49, 57, 58, 60], four quantitative [19, 51, 52, 55] and two MMs studies [22, 61].

(i) **Lack of family support in seeking PHC services**

Findings of the review disclosed that lack of cooperation from family members or relatives compelled the rural elderly to seek PHC services from their nearby places or persons, such as unqualified medical practitioners, village doctors, pharmacists, quacks, and traditional healers [22, 51, 52, 55, 60]. For example, the absence of family members support prevents the elderly from receiving PHC services from a qualified physician [51].

(ii) **Influence of family members on making healthcare decisions**

Three qualitative [18, 57, 58] and three quantitative studies [19, 51, 52] highlighted that the elderly were dependent on family members for two reasons: first, choosing the appropriate type of healthcare services, and second, meeting healthcare expenses. For example, Islam et al. (2022) denoted that a major portion of the elderly in the rural areas could not avail themselves of the institutional healthcare facilities as they needed to rely on the decision of their family members about selecting the pattern of PHC services [52]. Furthermore, it was also found that most of the rural elderly had to depend on their sons or daughters or relatives for their healthcare expenses and place of illness treatment [51].

(iii) **Family restrictions on receiving outside healthcare services for the elderly women**

Three qualitative studies [49, 58, 60] and an MMs study [61] disclosed that the female rural elderly faced several challenges from families to access healthcare services, such as a lack of mobility opportunities and strict adherence to religious practices. For example, due to religious restrictions on women's movement, most family members preferred to arrange treatment for the elderly female at home by a village doctor or traditional healer rather than taking them to outside qualified or skilled HPs [61].

#### Theme 3: Organizational-level barriers to accessing and using PHC services by the rural elderly.

The theme of organizational-level barriers included four subthemes: (i) inadequate internal facilities, (ii) maltreatment of HPs, (iii) preference to the gender of HPs, and (iv) high expense of treatment. The theme included eight studies, of which four were qualitative [11, 18, 49, 56], three were quantitative [19, 51, 53] and one was MMs study [22].

(i) **Inadequate internal facilities**

This subtheme included three quantitative [19, 51, 53] and one qualitative study [56]. The review highlighted that the scarcity of infrastructural facilities, including inadequate hospital beds, lack of diagnostic equipment, poor hygiene management, and absence of separate care units for the elderly, were the predominant barriers to accessing healthcare services by the rural elderly from the PHC facilities [51, 53, 56]. It was also found that managerial limitations, such as delayed service provision, non-availability of required medicine, lack of geriatric illness experts, lack of experience of the newly recruited HPs, and misconduct of service providers, were also the barriers to accessing and using PHC services by the rural elderly [19, 22, 53].

(ii) **Maltreatment of HPs**

Maltreatment of HPs utilizing government-run PHC services by the rural elderly was reported in three studies, including one MMs [22], two qualitative [18, 60] and one quantitative [53]. This maltreatment included the poor attitude of HPs and a lack of patience in hearing about all health problems [18, 22, 53]. This maltreatment of the HPs created mistrust on PHC services among the rural elderly, especially the elderly women [60]. As a result, some of the elderly reported that they were bound to visit private practitioners rather than the government-facilitated healthcare centers [53].

(iii) **Preference for the gender of HPs**

A notable barrier, reported in two qualitative studies [11, 56], to accessing PHC services by the rural elderly was the gender of physicians. For example, the rural elderly women preferred to seek healthcare services from female healthcare practitioners as they felt reluctant to share their health problems with male HPs due to prevailing social norms [56].

(iv) **High expense for treatment**

Two qualitative [18, 49] and two quantitative studies [19, 53] illustrated that the cost of treatment, including the expense of prescribed medicine and diagnosis costs, was a noticeable barrier to using PHC services by the rural elderly. Consequently, the rural elderly found that the alternative ailment treatment was less costly, such as homeopathic treatment or remedies from traditional healers [19].

#### Theme 4: Social and community-level barriers to accessing and using PHC services by the rural elderly.

To highlight social and community-level barriers to accessing and using PHC services by the rural elderly, two subthemes were identified: (i) sociocultural and (ii) geographical barriers. This theme included eight studies, of which seven were qualitative [11, 56–60, 62] and one MMs [61].

(i) **Sociocultural barriers**

In the four qualitative studies [11, 58, 60, 62], it was found that beliefs associated with illness, a culture of restriction, social taboos (e.g., concealing illness) regarding illness and ailment treatment were the dominant sociocultural barriers for the rural elderly to access PHC services, especially for elderly women. Mostly, the rural elderly women preferred to seek PHC services from nearby pharmacies, village doctors or traditional healers due to existing social beliefs and taboos regarding women’s health and illness [11, 56, 59, 61]. Consequently, religious healing chants, traditional healing systems, and self-medication or home-based cures were generally preferred by the elderly women to cure illness in rural areas [60, 61]. Social stigma (e.g., the prevalence of male dominance in making decisions about healthcare services) and religious restrictions (e.g., *purdah)* were also responsible for the lagging of the elderly women in accessing modern PHC services [11, 60]. One of the leading causes of the rural elderly’s dependency on the conventional healthcare system included easy accessibility, cost-effectiveness, overreliance on traditional healers, and not getting satisfactory accompany and motivational support from the neighbors [57, 58].

(ii) **Geographical barriers**

Two qualitative studies [57, 62] and one MMs study [61] revealed the geographical barriers to accessing and using PHC services by the rural elderly. The findings illustrated that distance to healthcare facilities and non-availability of transport, especially during monsoon, were also responsible for the rural elderly’s reluctance to seek quality PHC services [57, 61, 62]. It has also been reported that the rural aged women were reluctant to seek clinical PHC services due to the long distance to PHC centers and broken roads. In the monsoon, the roads become muddy and slippery, and it becomes hard to reach the healthcare center due to scarcity of transportation [61]. Consequently, the rural elderly had to rely on traditional home-based treatment or village doctors for treatment [62]. Additionally, this review also disclosed that the elderly did not get any healthcare support from their neighbors of the community, which delayed timely illness treatment in PHC centers [60].

#### Theme 5: Policy-level barriers to accessing and using PHC services by the rural elderly.

The policy-level barriers included two subthemes: (i) absence of health insurance, and (ii) inadequate health coverage. This theme consisted of seven studies, including five qualitative [11, 18, 56, 59, 60], one quantitative [51], and one MMs [61].

(i) **Absence of health insurance**

This subtheme was included in two qualitative studies [18, 60], one quantitative study [51], and one MMs study [61], where it is reported that the National Health Policy of Bangladesh 2011 lacks health insurance for the elderly. Therefore, the elderly’s inability to obtain and use PHC services was partly caused by a lack of legal framework and inadequate aged healthcare support [11, 56].

(ii) **Inadequate healthcare coverage**

Inadequate healthcare coverage denotes the insufficiency of the availability, accessibility, and affordability of quality healthcare services [63]. Inadequate health coverage for elderly people has been focused on as a policy barrier in three qualitative studies, one quantitative study [51] and one MMs study [61]. In this review, the key issues included in the inadequate health coverage were limited access to PHC services, high cost of healthcare services, scarcity of organizational resources, and crisis in the health workforce addressed by MoSW [23]. It was also reported that the shortage of trained HPs associated with scarcity of medicine supply resulted in lack of accessing healthcare services by the elderly women [61]. These issues are included in the PHC service centers, such as CCs, USCs, RSCs, and UHC, which provide special care to children and pregnant women compared to the elderly due to existing policies and regulations in the national health policy [18, 51, 59]. This is because the existing policies also lack proper attention to providing free PHC facilities for the elderly [51]. Consequently, the rural elderly experienced inadequate healthcare services due to lack of policy attention to their specific health needs [56].

## Discussion

This systematic review with thematic synthesis focused on the barriers to accessing and using PHC services by the rural elderly in Bangladesh. The barriers to accessing and using PHC services were identified and classified into five levels based on the SEM: (i) the individual, (ii) the family, (iii) the organizational, (iv) the social and community, and (v) the policy levels. The key findings at multiple layers of the SEM, identified from this review and supported by the previous literature, are discussed below, which are expected to be helpful for developing interventions to increase access to quality PHC services for the rural elderly and the development of policies in this area.

According to the findings at the individual-level obstacles, demographic barriers, such as growing age reduce the physical, mental and biological ability of the rural elderly and restrict their usage of PHC services. Similar findings have been observed in other studies [64–67]. The reason signified that, with the growth of age, the physical fitness of the elderly decreases because of frequent suffering of ailment and incapability of pursuing quality treatment [64]. As a result, they prefer to seek PHC services from nearby health practitioners [21, 68]. Additionally, married elderly were found more inclined to seek PHC services than those who did not have spouses, as the widowed or separated elderly experienced more lonliness than the married elderly, hardly had any emotionally close caregiver, and felt reluctant to seek PHC services [69].

Therefore, it was found that illiteracy hinders the rural elderly’s access to PHC services as they were less informed about the PHC services and was consistent with research findings from other South Asian nations [69–72]. However, illiteracy created knowledge gaps regarding healthcare service information among the rural elderly [73, 74]. Tefra et al. (2019) also reported that the illiterate elderly were burdened with illness as they could not afford the healthcare expenses on their own and were deprived of PHC services [73]. Furthermore, being female was reported as a barrier to seeking PHC services. Female elderly were more likely to be deprived of access to PHC services than their male counterparts due to gender orientation, structural barriers as well as patriarchal influence, and the finding was consistent with other studies conducted in developing countries [65, 72, 75, 76]. Consequently, elderly women faced a lack of awareness and social mobility, exhibit more submissiveness to reveal their illness, and often feel an inferiority complex to access PHC services [75, 77].

Another notable individual-level barrier was the financial insolvency of the rural elderly, which restricted their access to quality treatment from medical practitioners. The finding was associated with the studies conducted in India [70, 76] and Ethiopia [73]. The reasons behind the financial crisis for seeking treatment might be not having own income, getting no allowance, financial insecurity in families, and a lack of good relationships with relatives and peers and not having financial assistance from them [66, 78, 79]. As a result, they were unable to afford regular health check-ups and necessary medicines [73, 79]. Furthermore, in this review, knowledge gaps about medical services and treatment methods were identified as challenges in the uptake of primary health center services for rural adults, which is consistent with research conducted in other countries [78, 80–82]. The elderly in rural areas were less familiar with the accessibility and availability of existing PHC services and experience impediments to seeking PHC treatment due to a lack of shared health-related information by their FMs or community [80].

Key findings regarding family-level barriers included a lack of support from family members for the elderly to seek PHC services. The finding was consistent with other studies [4, 66, 70, 78]. Due to busy workdays or work pressure, family members might face difficulties managing time to accompany the elderly seeking care [78]. Another reason might be the increase in the number of single-parent families, the dependence on caregivers for physical and mental well-being, the feeling of burdening family members, and the lack of emotional closeness and increased communication gaps with relatives [4, 70, 83–85]. Moreover, the lack of decision-making ability to seek PHC services by the rural elderly in families was also identified in this review. The elderly lacked an independent income source to bear healthcare expenses and had to rely on others for healthcare costs, which was in line with studies conducted in developing countries [79, 83, 85–87]. For example, in India, it had been found that the comprehensive healthcare of the rural elderly depends on their family members and their treatment-seeking decisions due to economic overdependency on family [86]. Also, familial perception regarding gender and access to PHC services was also identified as a barrier in this review. The elderly women were less likely to seek PHC services than the elderly men due to cultural construction of gender, which was consistent with other studies conducted in South Asia [65, 86, 88]. For instance, Naz et al. (2021) disclosed that in Pakistan, due to religious restrictions (e.g., Purdah system), in families, the elderly women were not allowed to seek healthcare services alone [88].

The key organizational-level barrier included the lack of infrastructural and managerial facilities for the rural elderly created a hindrance to using PHC services. The findings were also associated with other studies conducted in Asia and African countries [4, 64, 66, 76–79, 84, 86]. Lack of infrastructural and managerial facilities include lack of treatment venues (e.g., public or private healthcare centers), limited choice of physicians, limited long-term care service options, untrained physicians, need for more medical service providers, difficulty in scheduling, and lack of trust in HPs [66, 78, 84]. The review also showed negligence of the HPs and lack of attention to the elderly also reduced the acceptance of formal PHC services. The finding identified in this review appeared consistent with the other studies [4, 75, 78, 79]. Adhikari et al. (2024) included that the hospital had a less friendly environment for the elderly because health workers judged them as elderly people and no one treats them satisfactorily [4].

Another important barrier at the organizational-level, identified in the review, was preference for the gender of healthcare professionals or providers at healthcare facilities to seek treatment of the rural elderly, especially by elderly women, which was consistent with other studies [14, 89–91]. The reason notified that due to invasive procedures, social traditions, social norms, social sanctions, and self-sanctions anticipate the gender of the HPs affecting the doctor-patient relationship [89, 91]. Furthermore, evidence suggested that female HPs were supposed to be more empathic and supportive to disseminate supplementary information regarding healthcare services [91]. On the other hand, some studies indicated that most of the time patients did not have a preference regarding the gender of the medical practitioners due to avail quality treatment [92–94]. Furthermore, another barrier was the lack of financial capacity to cover the costs of treatment, such as medicine and tests for the elderly, which was in line with studies conducted in other countries [64, 66, 76, 78, 84, 86]. Excessive treatment costs burden both the rural elderly and their families [64, 66, 86], discouraged them from seeking care.

The social and community-level barriers included that the patriarchal influence and dependency on male family members become an impediment to utilizing PHC services by aged women in Bangladesh [88, 95]. The structural orientation of patriarchy molded elderly women to take home-based care that significantly hindered their access to PHC services [88]. Furthermore, it was also found that prevailing stigma and social taboos regarding health and illness were also responsible for hindering access to PHC services by the rural elderly. Due to cultural constructions regarding illness, for example, declining health with age was normal, and the elderly hardly revealed their illness with others in fear of negative community perception, which aligned with other conducted studies on healthcare seeking behavior of the elderly [75, 96] Additionally, preference for ritualistic healers was also very common among the elderly of rural areas in India because of the reasonable rates and the additional benefit of receiving consultations at their homes [75]. Furthermore, in Ethiopia, it was found that the elderly of the countryside are less likely to seek PHC services due to lack of social ties, such as lack of interaction with neighbors, neighborhood support during illness, and deliberate disengagement in social networks [83, 84]. In contrast, Adhikari et al. (2024) found that sometimes the ill the elderly got social support from neighbors than their offspring [4].

Also, the influence of geographical barriers to accessing PHC services by the rural elderly at the social and community-level had been identified in this review. This barrier included the problem of long travel time, long distance, and inadequate transportation facilities to utilize formal PHC services by the elderly living in rural areas. The findings of this review aligned with the studies carried out in India, Nepal, Ghana, and Ethiopia [4, 73, 76, 78, 84]. Due to physical inability and lack of self-confidence, the rural elderly could not travel for a long time to reach the healthcare facility [4]. However, the challenge of distance was associated with a lack of public transportation for the elderly and their caregivers because of poor road and communication facilities along with high transportation costs in rural areas of developing countries [76]. For example, due to the lack of accessible roads, some of the elderly lost their lives on the way to the hospital [84]. Therefore, the elderly feel reluctant to visit the physicians office due to the long distance in the countryside of the USA [97].

Finally, in the policy-level barrier, the key findings of this review included that the lack of health insurance for the elderly in the national policy threatened access to PHC services. Similar findings were observed in other studies [98–102]. Due to financial constraints, the elderly and their caregivers feel the necessity of government assistance for healthcare support [100]. However, the rural elderly find it challenging to access high-quality services in PHC facilities since they lack government-sponsored health insurance or a health card [99]. Furthermore, a lack of sufficient health coverage for the elderly was also identified as a policy-level barrier in this review. Absence of adequate healthcare policy for the elderly was also noticed in the studies conducted in India, Sri Lanka, and Nepal [68, 81, 101, 103]. For instance, in India, inadequate aged care support and insufficient specific policy for monitoring disbursement of medical funds created barricades to utilizing PHC services by the rural elderly [81].

### Strengths and limitations

Studies of different research approaches, such as quantitative, qualitative, and MM studies, were included in this review, which provided a generalized notion about the barriers to access PHC services by the rural elderly in Bangladesh. Another strength of the study was that in the findings section, the information presented was based on the studies of rural areas of Bangladesh, but the discussion section compares it with the data from global perspectives, including the perspectives of South Asian countries, LMICs, developing countries, and underdeveloped countries. So, this review will be helpful for the researcher to conduct further studies related to this study and policy formation at the national level. Finally, the application of SEM helped to categorize the findings related to barriers from multiple perspectives.

However, like other review research, this current one has some limitations as well (i) the review focused on studies conducted in rural Bangladesh, which influenced selection bias, and restricted its applicability to urban Bangladesh and developed countries; and (ii) this review included only peer-reviewed published articles, which might limit the understanding of information available in report, conference proceedings, newspaper articles and generated publication bias, (iii) among 19 included studies in this review, 9 studies (47.37%) came from Hamiduzzaman et al. (6 studies) and Hossen & Westhues et al. (3 studies), which limits the generalizability of the findings with same participants and similar methods, and (iv) nearly half of the included studies (n = 9) focused exclusively on elderly women, resulting in an intensive emphasis on the barriers they face in accessing and using PHC services.

### Policy recommendations

Based on the review’s findings, the following recommendations, aligned with the SEM layers, can be implemented to enhance access to PHC services for the rural elderly in Bangladesh and other countries facing comparable challenges:

(i) At the individual level, increasing awareness of PHC services among the rural elderly through campaigns can be effective in accelerating their access to PHC services. Moreover, gender-sensitive health promotion policies, especially for elderly women, can be promoted by developing health literacy through community-based health literacy programs.
(ii) At the family-level, it is suggested to encourage FMs to strengthen the elderly’s freedom to take healthcare decisions and financial security, accompanying support and increase emotional closeness with the elderly to increase the access to PHC services timely through proper implementation and monitoring of ‘Bangladesh’s Parent’s Care Act 2013’ [24].
(iii) At the organizational level, gender-sensitive PHC delivery must be ensured, especially for elderly women. Therefore, the rural elderly’s access to PHC service centers can be increased by implementing regular managerial assessment systems and ensuring accountability among PHC service providers. Furthermore, initiating a healthcare coverage approach similar to India’s *Rashtriya Swasthya Bima Yojana (RSBY)* can minimize the organizational challenges [104].
(iv) To lessen social and community-level barriers, programs such as ‘The Community Health System (CHS)’ of Tanzania can be adopted to increase community support (e.g., neighborhood integrity, community volunteers, support from the local leaders, etc.) [105], and strengthen awareness regarding PHC services among the rural elderly. Findings of the review also suggested normalizing the ageing process by community-based health literacy programs, especially for the elderly women.
(v) At the policy level, initiating health insurance for the rural elderly will facilitate their access to PHC services without financial burdens. Health insurance has proven effective in India, where the government has initiated the *Ayushman Bharat* health insurance policies specifically for individuals aged 60 and above [106]. This initiative empowers the elderly and diminishes their reliance on FMs.

## Conclusions

The systematic review with thematic synthesis, which included a systematic search, has addressed the multilayered barriers that the rural elderly in Bangladesh face when accessing and using PHC services. The SEM was used to summarize a wide-ranging multilevel barrier to addressing the rural elderly’s access and use of PHC services at individual, family, organizational, social and community, and policy-levels. Findings disclosed individual-level barriers, including financial insolvency, dependency, and lack of knowledge related to PHC services. Additionally, family-level barriers included lack of communication with FMs and relatives and lack of control over family decisions, which hindered rural elders from accessing quality PHC services. Moreover, poor infrastructural facilities, the attitude of HPs, and the elderly women’s preference for the gender of the HPs appeared as key barriers at the organizational-level. Also, social and community-level barriers involved overdependency on traditional or self-medicines and difficulties reaching the PHC service centers. Finally, policy-level barriers reported a lack of awareness-building strategies and a proper health policy for the rural elderly in this review. However, based on the findings of the review, it is essential to reduce the knowledge gap in PHC services, increase family support, improve the healthcare infrastructure and managerial facilities, raise community awareness through camping or courtyard meetings with community people, and initiate healthcare programs for the elderly.

## Supporting information

S1 File Systematic review protocol. (PDF)

S2 File PRISMA 2020 checklist. (DOCX)

S1 Table Mapping themes, subthemes, and codes of the study with the SEM framework. (DOCX)

## Abbreviations

CCs: Community Clinics
CASP: Critical Appraisal Skills Programme
FGs: Focus Groups
GoB: Government of Bangladesh
HBM: Health Belief Model
HPs: Healthcare Providers
IDIs: In-depth Interviews
KIIs: Key Informant Interviews
LMICs: Low- and Middle-Income Countries
MOHFW: Ministry of Health and Family Welfare
MoSW: Ministry of Social Welfare
MMAT: Mixed Methods Appraisal Tool
MMs: Mixed Methods
PHC: Primary Healthcare
PEO: Population, Exposure and Outcome
PRISMA: Preferred Reporting Items for Systematic Reviews and Meta-Analyses
RSC: Rural Sub-centers
SDG3: Sustainable Development Goal 3
SDGs: Sustainable Development Goals
SEM: Social Ecological Model
UHC: Upazila Health Complex
UN: United Nations
USCs: Union sub-centres
WHO: World Health Organization
