# Supplementary material for: Using the Social Ecological Model to identify barriers to accessing and using primary healthcare services by the rural Bangladeshi elderly: A systematic review
Source: PLoS One. 2026 Sep 18;21(9):e0358477. doi: 10.1371/journal.pone.0358477 (PMC13588402; doi:10.1371/journal.pone.0358477)
Supplement: Supplementary file 1 [file pone.0358477.s001.pdf]

Systematic Review Protocol

1. Title

Using the Social Ecological Model to Identify Barriers to Accessing and Using Primary Healthcare Services by the Rural Bangladeshi elderly: A Systematic Review

2. Review Question

What are the barriers influencing access to and use of primary healthcare (PHC) services among rural elderly populations in Bangladesh?

3. Background

Bangladesh's government is focusing on the United Nations Sustainable Development Goal 3 to improve primary healthcare (PHC) services, although rural elderly people still lack access. However, no systematic review has yet synthesized the available evidence on the factors shaping access to PHC among the rural elderly in Bangladesh. This review aims to fill this gap by systematically identifying, appraising, and synthesizing published and grey literature on the topic.

4. Aim

The systematic literature review aimed at exploring and synthesizing the multilevel barriers to accessing and using PHC services by the rural Bangladeshi elderly based on the Social Ecological Model (SEM).

5. Eligibility Criteria

Criteria	Inclusion criteria	Exclusion criteria
**Text**	Full text peer-reviewed studies	Conference paper, abstract, non-peer reviewed studies and newspaper articles
**Research method**	Qualitative, Quantitative and Mixed Methods	Review
**Aim**	Studies focusing on access and use of PHC or healthcare services and barriers of PHC and healthcare services	Studies highlighting secondary and tertiary healthcare access and utilization
**Age**	60 and above years old	Below 60 years old
**Participants**	Elder people (both male and female)	Children, adolescents and adult people
**Region**	Rural areas of Bangladesh	Developed countries, least developed countries and only urban areas
**Language**	English	Bangla and other languages
**Limit of duration of publication**	Studies published in the year of 2000 to 2024	Studies published before the year of 2000

6. Information Sources

Databases: PubMed, Scopus, Proquest, Google Scholar, and Google

7. Data Management and Selection Process

Two reviewers will independently screen titles, abstracts, and full texts using predefined inclusion criteria. Discrepancies will be resolved through consensus or a third reviewer. The PRISMA 2020 flow diagram will be used to report the selection process.

8. Data Extraction

Data extraction will include: author, year, study design, sample, pattern of seeking healthcare by the elderly, and barriers oto accessing PHC services by the rural Bangladeshi elderly.

9. Quality Assessment

Critical Appraisal Skills Programme (CASP), adopted from Crombie for quantitative study, Critical Appraisal Skills Programme (CASP) for qualitative study, and Mixed Methods Appraisal Tools (MMAT) for MMs study to assess the quality

10. Data Synthesis

Thematic analysis, following the phases outlined by Thomas and Harden (2008), will be employed to synthesize the study's results. The themes will be organized deductively, as mentioned in the SEM model, to identify the barriers to accessing PHC services by the rural elderly. The characteristics of the included studies will be summarized and tabulated following the guidelines of Ma et.al. (2017).

11. Registration and Reporting

This review will be registered in PROSPERO (registration number to be assigned) and follow PRISMA 2020 guidelines.

12. Dissemination Plan

Findings will be published in peer-reviewed journals

13. Review Team

Principal Investigator:

Dipika Chandra, Khulna University. Bangladesh. Email: dipika09@soc.ku.ac.bd

Co-Authors:

Mr Sanjoy Chanda. Khulna University. Bangladesh. Email: skchanda@soc.ku.ac.bd

No conflict of interest declared.

Mr Tunvir Shohel. Khulna University. Bangladesh. Email: tunvirshohel@soc.ku.ac.bd

No conflict of interest declared.

Mr Bijoy Banik. University of Rajshahi. Bangladesh. Email: bkbanik2001@ru.ac.bd

No conflict of interest declared.

Ms Vinami Yulian. Universitas Muhammadiyah Surakarta. Indonesia. Email: vinami.yulian@ums.ac.id

14. Funding

No external funding.

15. Conflicts of Interest

None declared.

16. Timeline

Protocol development: October 2025
Literature search: October 2025
Screening: October 2025
Analysis: November 2025
Manuscript writing: December 2025

17. Keywords

Barriers and facilitators; Healthcare access; Primary healthcare; Rural areas; Bangladesh
