# Supplementary material for: Using the Social Ecological Model to identify barriers to accessing and using primary healthcare services by the rural Bangladeshi elderly: A systematic review
Source: PLoS One. 2026 Sep 18;21(9):e0358477. doi: 10.1371/journal.pone.0358477 (PMC13588402; doi:10.1371/journal.pone.0358477)
Supplement: Supplementary file 3 [file pone.0358477.s003.docx]

**Mapping themes, subthemes, and codes of the study with the SEM framework**

| **SEM levels** | **Theme** | **Subtheme** | **Code** | **Evidence** |
| --- | --- | --- | --- | --- |
| **Intrapersonal** | Individual level barriers | Demographic barriers | -Age  -Gender  -Not having a spouse  -Living alone  -Illiteracy  -Decline of physical ability  -Gaps in knowledge | Abdullah et al., 2018; Akter et al., 2023; Biswas et al., 2006; Hamiduzzaman et al., 2021a; Hamiduzzaman et al., 2021b; Hamiduzzaman et al., 2022a; Hamiduzzaman et al., 2022b; Hamiduzzaman, 2020; Hossen & Westhues, 2010; Hossen & Westhues, 2011; Hossian et al., 2019; Islam et al., 2022; Jabeen et al., 2015 |
|  |  | Financial insolvency | -Not having money for treatment  -Lack of independent healthcare decision-making capability |  |
|  |  | Knowledge gap regarding PHC services | -Lack of awareness  -Lack of health-related information |  |
| **Interpersonal** | Familial level barriers | Lack of family support in seeking PHC services | -Lack of cooperation | Abdullah et al., 2018; Biswas et al., 2006; Biswas et al., 2007; Hamiduzzaman, 2020; Hamiduzzaman et al., 2021b; Hamiduzzaman et al., 2022a; Hossen & Westhues, 2010; Hossian et al., 2019; Islam et al., 2022; Sarker et al., 2023 |
|  |  | Influence of family members on making healthcare decisions | -Overdependence of family members on healthcare decisions  -Dependence on family members for healthcare expenses |  |
|  |  | Family restrictions on receiving outside healthcare services for the elderly women | -Lack of mobility opportunities  -Strict adherence to religious practices |  |
| **Institutional** | Organizational level barriers | Inadequate internal facilities | -Scarcity of infrastructural facilities  -Managerial limitations | Abdullah et al., 2018; Biswas et al., 2006; Biswas et al., 2007; Hamiduzzaman et al., 2021a; Hamiduzzaman et al., 2022a; Hamiduzzaman et al., 2022b; Hossian et al., 2019; Jabeen et al., 2015; Sarker, et.al., 2023 |
|  |  | Maltreatment of HPs | -Poor attitude of HPs  Lack of patience in hearing about all health problems |  |
|  |  | Preference to the gender of HPs | -Gender of physicians |  |
|  |  | High expense for treatment | -Cost of treatment |  |
| **Community** | Social and community level barriers | Sociocultural barriers | -Beliefs associated with illness  -Culture of restriction -Social stigmas and taboos regarding illness and ailment treatment  -Belief in traditional healing methods | Hamiduzzaman, 2020; Hamiduzzaman, et al., 2021a; Hamiduzzaman, et al., 2021b; Hamiduzzaman et al., 2022a; Hamiduzzaman et al., 2022b; Hamiduzzaman, 2020; Hossen & Westhues, 2010; Hossen & Westhues, 2011; Hossen & Westhues, 2012 |
|  |  | Geographical barriers | -Distance to healthcare facilities  -Non-availability of transport  -Broken roads  -Unfavorable weather condition |  |
| **Policy** | Policy level barriers | Absence of health insurance | -No separate geriatric health insurance policy | Biswas et al., 2006; Hamiduzzaman et al., 2021a; Hamiduzzaman, 2022a; Hamiduzzaman et al., 2022b; Hamiduzzaman, 2020; Hossian et al., 2019; Hossen & Westhues, 2011 |
|  |  | Inadequate health coverage | -Limited PHC services for the elderly  -High cost of healthcare services  -Scarcity of organizational resources  Crisis in the health workforce addressed |  |
