# Supplementary material for: Using the Social Ecological Model to identify barriers to accessing and using primary healthcare services by the rural Bangladeshi elderly: A systematic review
Source: PLoS One. 2026 Sep 18;21(9):e0358477. doi: 10.1371/journal.pone.0358477 (PMC13588402; doi:10.1371/journal.pone.0358477)
Supplement: Supplementary file 4 [file pone.0358477.s004.docx]

Dear Editor,

We sincerely thank you for your thoughtful and constructive feedback on our manuscript. We have carefully revised the paper in line with the comments. All substantive revisions made in response to the comments have been placed in the revised manuscript and are explained in detail in the point-by-point responses in Table 1 below.

Table 1. Point-by-point response

| **Comments** | **Authors’ response** |
| --- | --- |
| Editor |  |
| - In the case of your submission, we note that language restrictions are imposed on the literature searches without adequate justification. There is increasing recognition that exclusion of studies published in other languages can increase the risk of bias in systematic reviews. We feel that any restrictions should be justified based on scientific reasons rather than resource constraints. As a result, we have concerns about the comprehensiveness of the review and the validity of the conclusions. | - Thank you for your comments. As per your suggestion, without language restrictions on literature, a new search has now been undertaken with three electronic databases, including Scopus, PubMed, and ProQuest Sociological Abstracts, and a manual search has been conducted with Google Scholar and Google in March 2026. The new searches have returned 3 new articles, which have now been added with previous articles. In addition, exclusion and inclusion criteria, search strategies, critical appraisal tables, characteristics tables, PRISMA flowchart, and results have now been revised. Please see the revised version of the manuscript. |
