# Supplementary material for: Using the Social Ecological Model to identify barriers to accessing and using primary healthcare services by the rural Bangladeshi elderly: A systematic review
Source: PLoS One. 2026 Sep 18;21(9):e0358477. doi: 10.1371/journal.pone.0358477 (PMC13588402; doi:10.1371/journal.pone.0358477)
Supplement: Supplementary file 5 [file pone.0358477.s005.docx]

Dear Editor,

We would like to appreciate you for your valuable comments along with additional editor and reviewers on the manuscript (Reference no. PONE-D-26-12917)**.** We have considered all the comments seriously and have updated the manuscript according to the comments.

The point-by-point response file has been included below for your consideration.

Thank you,

The corresponding author

**Response to the Editor**

| Sl. no. | **Editor’s comment** | **Authors’ response** |
| --- | --- | --- |
| 1. | Please ensure that your manuscript meets PLOS ONE's style requirements, including those for file naming. | Many thanks for your comment. We have ensured that our manuscript meets PLOSE ONE’s style requirements. |
| 2. | If the reviewer comments include a recommendation to cite specific previously published works, please review and evaluate these publications to determine whether they are relevant and should be cited. There is no requirement to cite these works unless the editor has indicated otherwise. | Thanks for your advice. |

**Response to the Additional Editor**

| Sl. no. | **Additional Editor’s comment** | **Authors’ response** |
| --- | --- | --- |
| 1. | - **Screening Rigor and Study Inclusion/Exclusion (Critical) Inclusion of Study 10**   Reviewer 1 rightly points out that Study 10 (Hamiduzzaman et al., 2022a) includes zero elderly participants (only healthcare professionals). Since your review specifically targets barriers faced by the rural elderly themselves, the inclusion of this study represents a serious screening error. You must re-evaluate your screening process, exclude this study (and any others failing the age eligibility criteria), and update your PRISMA flowchart, analysis, and synthesis accordingly. | - Many thanks for raising the issue. As suggested, study 10 (Hamiduzzaman et al., 2022a) has now been excluded from study, updated the PRISMA flowchart, analyzed and synthesized accordingly. Please see Tables 3 and 4 of the tracked-changes version of the manuscript and Figure 2. |
| 2. | - **Overlapping Sample Cohorts and Independence of Evidence** A significant proportion (45%) of your included studies originate from only two author groups (Hamiduzzaman; Hossen & Westhues), focused on very similar samples in the Sylhet district. Treating these as entirely independent sources of evidence inflates the weight of your qualitative synthesis. You must explicitly address this limitation, discuss the potential double-counting/overlap of study cohorts, and temper your conclusions. | - Thanks for your comment. The authors agree with you that most of the studies on the elderly in Bangladesh have been conducted by Hamiduzzaman et al; Hossen & Westhues basically. As you suggested, the authors have addressed this issue in the limitations section of the manuscript. Please see the limitations section of the tracked-change version of the manuscript (lines 666-668). |
| 3. | **Theoretical Framework and Conceptual Accuracy**   - Origins of the SEM: Correct the attribution of the Social Ecological Model. Lines 111-112 attribute the model to Bronfenbrenner (1977), which is actually the Ecological Systems Theory from developmental psychology. The public health-specific SEM used in your study was popularized by McLeroy et al. (1988). - Andersen’s Behavioral Model: Your critique of Andersen’s model focuses solely on its original 1968 formulation. However, Andersen revised this framework in 1995 (and subsequently) to explicitly incorporate contextual, cultural, and environmental factors. Please update this conceptual critique to reflect contemporary versions of the model. | - Many thanks for your comment. As suggested, in the guiding framework section, McLeroy et al. (1988) have now been credited for popularizing SEM in public health. Please see lines 122-126 of the tracked-changed manuscript. - Furthermore, the updated version of Andersen’s Behavioral Model has been taken into account for reasoning the use of SEM as the theoretical ground of this study. Please check the tracked-changed version of the manuscript (lines 116-119). |
| 4. | - **Quality Appraisal Methodological Flaws** - Appraising quality using arbitrary percentages (e.g., scoring CASP and MMAT out of 100%) is highly discouraged by Cochrane and the respective tool developers, as it masks critical domain-specific flaws. Please revise your quality appraisal to present a domain-based risk-of-bias/appraisal assessment. Address the appraisal logical inconsistencies (e.g., rating a study with a sample size marked as "Not reported" as having "High quality" and full marks for recruitment and data collection). | Many thanks for your concerns. As suggested, we have now removed the scoring procedure and used the Cochrane three levels of risk-of-bias, including low, some concerns and high. Please see the revised quality assessment section (lines 251–257), the right column of Table 3, and the characteristics section (lines 291–295) in the tracked‑changes version of the manuscript. |
| 5. | - **Over-generalization and Gender Representation**   Half of your included studies (10 out of 20) focus exclusively on elderly women. However, your discussion, conclusion, and policy recommendations are framed as applying universally to all rural Bangladeshi elderly (male and female). Please contextualize your findings to acknowledge this strong female bias and restrict over-generalizations. | - Thanks for your comment. There are a few studies conducted on the elderly (both male and female) in Bangladesh. Consequently, the authors have included the studies conducted on elderly women only. This is a limitation of the study, which has been addressed in the limitations section. Please check lines 669-671 of the tracked-changed manuscript. Furthermore, the discussion, recommendations, and conclusions sections have been reworked, please see the tracked-changed version of the manuscript. |
| 6. | - **Transparency and Reproducibility of Search Strategy** - Please provide the full database-specific search strings used for each database to ensure reproducibility, as highlighted by Reviewer 4. - Verify and correct your PROSPERO registration ID (CRD420251164335A). Trailing letters in PROSPERO IDs are highly unusual and likely represent a transcription error. - Resolve the arithmetic and layout inconsistencies in your PRISMA flowchart (e.g., overlapping/concatenated numbers in the tracked changes version). | - Thanks for the comment. The authors have represented database-specific search strings (Boolean operators, truncation, and field tagging) in Table 2 of the track-changed manuscript (line 192). - Thanks for noticing this issue. The letter ‘A’ was mistakenly written with the registration number, and it has now been revised. Please check line 140 of the tracked-changed manuscript. - As suggested, the PRISMA flowchart has been revised. Please check Figure 2 of the tracked-changed version of the manuscript. |

**Response to the Reviewer 1**

| Sl. no. | **Comment Reviewer 1** | **Authors’ response** |
| --- | --- | --- |
| 1. | - PRISMA numbers don't match across documents. | - Thank you so much for your comment. The entire document has been rechecked by the authors, and the manuscript has been revised following the PRISMA numbers. Therefore, the PRISMA flowchart has been updated with 19 studies (Figure 2) as one study (Hamiduzzaman et al., 2022a) did not fulfil the eligibility criteria of the study participants. Please see Figure 2. |
| 2. | - **The SEM is credited to the wrong person:** Lines 111–112 say "The SEM developed by Bronfenbrenner (1977)." Bronfenbrenner developed Ecological Systems Theory — a child development model from psychology. The Social Ecological Model used in public health was developed by McLeroy et al. (1988). These are different frameworks from different fields. The SEM is the entire basis of this review. Getting its origin wrong is a foundational error. | - Many thanks for your comment. In the guiding framework section, McLeroy et al. (1988) have been credited for popularizing SEM in public health. Please check the tracked-changes manuscript (lines 122-125). |
| 3. | - **One included study has zero elderly participants:** The eligibility criteria (Table 1) require elderly participants. Study 10 (Hamiduzzaman et al. 2022a) includes only doctors (n=3), pharmacists (n=3), a health inspector (n=1), and health assistants (n=4). No elderly people. It should have been excluded at screening. It was instead rated 100% quality and included in the synthesis. | - Thanks for noticing the issue. The authors have now excluded Study 10 (Hamiduzzaman et al., 2022a) from the review (please check Table 4 of the current manuscript) and updated the PRISMA flowchart accordingly (Figure 2). |
| 4. | - **Publication year range is wrong:** Lines 273–275 say studies were published "between 2005 and 2026." The earliest study in Table 4 is from 2006 (Biswas et al.). No 2005 study exists. Also, the eligibility window starts at 2000, but nothing from 2000–2005 was found — and this six-year gap is never mentioned. | - Thanks for your comment. No relevant study was found for the period 2000-2005. Consequently, the authors could not include any study of this period. The studies found from the electronic and manual databases ranged from 2006 to 2025, which have been included in the manuscript. Please see lines 315-316 of the track-changed manuscript. |
| 5. | - **A sentence meant to justify the study design contradicts itself:** Lines 132–134 read: "a systematic review was deemed the most suitable approach...rather than merely summarizing findings in relation to a specific research question, as would be done in a systematic review." It justifies systematic review by contrasting it with systematic review. The word at the end should be "narrative review." This survived revision uncorrected. | - Thanks for noticing it. The sentence concerned has now been amended in the track-changed manuscript (lines 143-147). |
| 6. | - **45% of studies come from two author groups, with likely overlapping samples:** Hamiduzzaman authored 6 of 20 studies; Hossen & Westhues authored 3. Together: 9 of 20 (45%). Several Hamiduzzaman studies involve elderly women in Sylhet district with ~25 participants and similar methods — likely from the same or overlapping samples. Synthesizing these as independent evidence inflates the strength of findings. This is not acknowledged anywhere in the paper. | - Thanks for your comment. There are a few studies conducted on the elderly (both male and female) in Bangladesh. Consequently, the authors have included the studies conducted on elderly women only. This is a limitation of the study, which has been addressed in the limitations section of the track-changed manuscript (lines 666-668). |
| 7. | - **Every single study rated "High" quality:** All 20 studies scored High. All 7 quantitative studies received exactly 10/12 with identical Yes/No patterns — suggesting a template was applied, not genuine individual appraisal. Two studies with no ethical approval statement were still rated High. The quality classification method itself cites a paper by the corresponding author of this review (Reference 40, Chanda et al. 2022) — an unacknowledged self-citation as methodological authority. | - Many thanks for your concerns. We have now removed the scoring procedure and used the Cochrane three levels of risk-of-bias, including low, some concerns and high. Please see the revised quality assessment section (lines 251–257), the right column of Table 3, and the characteristics section (lines 291–295) in the tracked‑changes version of the manuscript. |
| 8. | - **One study has no reported sample size but gets a perfect quality score:** Study 12 (Table 4) lists sample size as "N= Not reported." Table 3 gives it 10/10 (100%) on the CASP qualitative checklist, including full marks for recruitment appropriateness and data collection adequacy. You cannot assess these without knowing how many participants there were. | - Thanks for noticing this. In Table 3 of the tracked-changed version of the manuscript, the quality of the concerned study (Hamiduzzaman et.al., 2021a) has now been rechecked, and the risk-of-bias of the study has been included instead of scoring. |
| 9. | - **Most studies are about elderly women, but conclusions claim to cover all elderly:** At least 10 of 20 included studies focus exclusively on elderly women. - The conclusions and all five policy recommendations (lines 598–620) are written as if they apply to all rural elderly in Bangladesh. - Claims comparing elderly men and women (lines 306–307) cannot be made from a literature base that barely includes elderly men | - Thank you for the comment. This issue has been identified in the Strengths and limitations section of the tracked-changed manuscript (lines 666-668). - As suggested, the policy recommendations and conclusions have been revised by the authors. Please see the current tracked-changed version of the manuscript (lines 673-708). - Thanks for noticing this issue. The sentence has been revised in the tracked-changes manuscript (lines 349-351). |
| 10. | - **PROSPERO ID has a non-standard trailing letter:** The registration number given is "CRD420251164335A." PROSPERO IDs never end with a letter. This may be a transcription error. Since the registration number must be publicly verifiable, it must be corrected to exactly what appears in the PROSPERO database. | - The letter ‘A’ was mistakenly written with the registration number. The authors have omitted the letter from the text. Please see the track-changed manuscript (line 140). |
| 11. | - **The critique of Andersen's model targets its 1968 version:** Lines 106–108 say Andersen's model ignores culture and social interaction. This was true of the 1968 original. Andersen revised the model in 1995 and again subsequently to explicitly include contextual factors, social environment, and cultural norms. A 2026 paper should engage with the current version of a competing framework, not a 58-year-old one. | - Many thanks for addressing the issue. The updated version of Andersen’s Behavioral Model has been taken into account for reasoning the use of SEM as the theoretical ground of this study. Please check lines 116-119 of the track-changed manuscript. |
| 12. | - **A finding with no supporting literature is presented without any caveat:** Lines 527–530 explicitly state: "no study was found focused on the rural elderly in line with the findings" — referring to gender preference for healthcare providers. Despite this admission, the finding is discussed at length and included in the conclusions (line 631) as if it has the same evidentiary weight as findings supported by multiple studies. It should be clearly labelled as preliminary and requiring further investigation. | - The authors have revised the sentence and included relevant citations regarding gender preference of the elderly in seeking PHC services in the Discussion section of the track-changed file (lines 587-594). |

**Response to the Reviewer 2**

| Sl. no. | **Comment of the Reviewer 2** | **Authors’ response** |
| --- | --- | --- |
| 1. | Given that the authors derive policy recommendations from primary data subjected to statistical analysis and meta-analytic synthesis, one might anticipate methodological rigor. However, the analytical framework exhibits critical limitations that undermine the validity and generalizability of both findings and prescriptive outcomes. Specifically, the statistical models appear to suffer from inadequate adjustment for confounding variables, potential selection bias in data aggregation, and insufficient sensitivity analyses to validate meta-analytic effect sizes. Consequently, the resultant policy recommendations lack the robustness required for evidence-based decision-making, rendering them vulnerable to overgeneralization and reduced translational impact in real-world policy contexts.  So I would strongly recommend to include meta-analysis in the study. | - Many thanks for your comments. We highly appreciate your comments. As this review included studies of multiple approaches, including quantitative, qualitative, and mixed methods, and did not use solely quantitative studies, the thematic synthesis approach has been found appropriate to synthesize data. Consequently, it is not now possible for the authors to include meta-analysis in the study. We apologize for that. We must consider your advice while we will develop future reviews with quantitative studies. |

**Response to the Reviewer 3**

| Sl. no. | **Comment of the Reviewer 3** | **Authors’ response** |
| --- | --- | --- |
| 1. | - The manuscript has a number of grammatical errors especially in the study design section. - While it is clear there is a need for this study, the authors did not do a thorough job in providing evidence for the significance of the study in the background section. - Finally, there are multiple communities and villages in rural Bangladeshi but the authors did not specify which communities were included in the review. | - Thanks for your comments. The authors have rechecked grammatical issues to minimize grammatical mistakes not only in the study design section but also across the document. - As suggested, the background section has now been revised. Please see the background section of the tracked-changed manuscript (lines 69-72 and 91-94). - The systematic review included published papers from the elderly living in mainstream rural Bangladesh in general and did not include any papers from hill tracks or marginalized communities, for this reason, it was not necessary to specify communities in rural Bangladesh. |

**Response to the Reviewer 4**

| Sl. no. | **Comment of the Reviewer 4** | **Authors’ response** |
| --- | --- | --- |
| **Major Comments** | | |
| 1. | - **Study Design and Conceptual Clarity** The manuscript presents an internal inconsistency in defining the study design. - Action: Please clearly define the study as a systematic review with thematic synthesis and ensure consistency across all sections | - Thanks for noticing the error. The sentence concerned study design, has now been amended in the track-changed manuscript (lines 143-147). - As suggested, the term systematic review with thematic synthesis has been reported in Data synthesis section (lines 286-288) along with the required sections of the tracked-changed manuscript. |
| 2. | **Search Strategy and Reproducibility** The search strategy is not reproducible.  What to do:   - Provide full database-specific search string - Specify search dates, language limits, and grey literature strategy - Define Google Scholar screening limits | - Thanks for the comment. The authors have represented database-specific search strings (Boolean operators, truncation, and field tagging) in Table 2 of the track-changed manuscript (line 192). - The search date of the literature has been mentioned in line 168 of the track-changed manuscript. Additionally, this review did not apply any language restrictions in order to minimize bias. Therefore, the reasons for not using grey literature in this review have been reported in the Eligibility criteria section (lines 153-160) of the revised manuscript. - As suggested, the Google Scholar screening limits are informed in Search strategies section of the track-changed manuscript (lines 183-185). |
| 3. | - **Application of the PICO Framework**   PICO is incorrectly operationalised. Authors may reframe PICO with Population (elderly), Exposure (barriers), Outcome (PHC access and utilization). | - As suggested, the PICO framework has now been replaced with PEO framework. Please see lines 174-179 of the track-changed manuscript. |
| 4. | - **Eligibility Criteria** PHC not clearly defined and timeframe unjustified. - Justify the time restriction | - Thanks for the comment. The definition of PHC is revised in the background section of the track-changed file (lines 69-72). In addition, no relevant study was found for the period 2000-2005. As a result, the authors have included papers ranging from 2006 to 2025. Please see the track-changed manuscript (lines 315-316). - As suggested, the justification of the time restriction (2000-2026) has been provided in lines 153-157 of the track-changed manuscript. |
| 5. | - **Study Selection Process** There is no inter-rater reliability reported. The authors should report agreement statistics or describe the consensus process(Cohen’s kappa). | - As suggested, inter-rater reliability was assessed using Cohen’s kappa (κ = 0.78, 95% CI 0.72–0.84), indicating substantial agreement. Please see lines 218-220 of the track-changed manuscript. |
| 6. | - **Quality Appraisal** There is an inappropriate scoring of CASP and MMAT. Authors may remove the scoring and present the domain-based risk-of-bias assessment. | - Many thanks for your concerns. As suggested, we have now removed the scoring procedure and used the Cochrane three levels of risk-of-bias, including low, some concerns and high. Please see the revised quality assessment section (lines 251–256), the right column of Table 3, and the characteristics section (lines 291-295) in the tracked‑changes version of the manuscript. |
| 7. | - **Data Synthesis** Why only Descriptive and deductive?      - Clarify coding approach - Strengthen analytical synthesis and compare findings across studies, and avoid isolated statistics. | - Thank you so much for noticing this issue. The explanation of using descriptive and deductive approach has been reported in the track-changed manuscript (lines 286-288). - As suggested, the clarification of coding approach has been provided in the revised version of the manuscript. Please check lines 291-295 of the manuscript. - According to your suggestion, the isolated statistics have been deleted from the Descriptive themes section of the track-changed manuscript. Furthermore, the analytical synthesis and comparison with findings across studies have been revised in Discussion section of this revised manuscript. |
| 8. | - **Conceptual Framing** There is a mischaracterisation of Andersen’s model. Authors should correct and provide balanced justification for SEM. | - According to your suggestion, the updated version of Andersen’s Behavioral Model has been taken into account for reasoning the use of SEM as the theoretical ground of this study. Please see the Guiding framework section of the track-changed manuscript (lines 116-119). |
| 9. | - **Discussion** The interpretation is limited under the discussion section. - Expand theoretical and policy implications. | - The Discussion section has now been revised by the authors. Please check the Discussion section of the track-changed file. - According to your suggestion, the Policy recommendations section has been revised. Based on findings and the theoretical framework, the policies have been suggested to increase access to PHC services by the rural elderly in Bangladesh. Please check lines 673-708 of tracked changed file. |
| 10. | - **Limitations** Include bias and methodological limitations. | - As suggested, pertinent bias and methodological limitations have been reported in the Strengths and limitations section of the track-changed file (lines 660-671). |
| **Minor Comments** | | |
| 1. | Standardise terminology (elderly vs older adults) | - The term elderly has been used throughout the manuscript. The term older adults has been reported in the Reference section only, which cannot be changed as they were already published. |
| 2. | Reduce repetition | - The revised version of the manuscript has been rechecked by the authors thoroughly, and the repetition has been deleted. |
| 3. | Correct grammatical inconsistencies | - The grammatical errors have been rechecked and corrected by the authors. |
| 4. | Remove duplicate abbreviations (e.g., HPs) | - As suggested, the duplication of using HPs has been removed. Please check lines 85 of the track-changed file. |
