# Supplementary material for: Using the Social Ecological Model to identify barriers to accessing and using primary healthcare services by the rural Bangladeshi elderly: A systematic review
Source: PLoS One. 2026 Sep 18;21(9):e0358477. doi: 10.1371/journal.pone.0358477 (PMC13588402; doi:10.1371/journal.pone.0358477)
Supplement: Supplementary file 6 [file pone.0358477.s006.docx]

Dear Editor,

We would like to appreciate you for giving the opportunity to address the minor editorial and typographical errors, along with the comments of additional editor on the manuscript (Reference no. PONE-D-26-12917)**.** We have considered all the comments seriously and have updated the manuscript according to the comments.

The point-by-point response file has been included below for your consideration.

Thank you,

The corresponding author

**Response to the Editor**

| Sl. no. | **Editor’s comment** | **Authors’ response** |
| --- | --- | --- |
| 1. | - **Subtheme Numbering (Theme 3):**   On page 36 (lines 402–404), the subthemes are currently numbered as "(i), (iii), (iii), (iv)". Please correct this sequence to "(i), (ii), (iii), (iv)". | - Many thanks for noticing the mistake. The sequence to "(i), (ii), (iii), (iv)" has now been revised. Please check line 372 of the revised tracked-change version of the manuscript. |
| 2. | - **Figure 2 Labeling:**   In the uploaded image file for Figure 2 (the PRISMA flowchart), the internal caption embedded within the image reads "Figure 1 PRISMA flowchart...". Please correct this inside the graphic to "Figure 2". | - Thanks for the comment. The figure number has now been corrected inside the image file. Please check the current version of the tif file of Figure 2. |
| 3. | - **Typographical Errors in Table 4:**   Row 1 (Sarker, 2025): Please correct "Corress-sectional survey" to "Cross-sectional survey".  Row 16 (Biswas et al., 2007): Please correct "Nnon-random strategy" to "Non-random strategy". | - Thanks for noticing the issue. The typo ‘Corress-sectional survey’ has now been replaced with ‘Cross-sectional survey’ in Row 1 of Table 4 (line 280) of the revised tracked-change version of the manuscript. - Many thanks for noticing the error. The typo ‘Nnon-random strategy’ has now been replaced with ‘Non-random strategy’ in Row 16 of Table 4 (line 280) of the revised tracked-change version of the manuscript. |
| 4 | - **Minor Spacing:**   On page 26, line 292, please add a space between the citation bracket and the following word ("[48] was followed"). | - Thanks for detecting the issue. A space has been added between the citation bracket and the following word. Please check line 271 of the revised tracked-change version of the manuscript |

**Response to the Additional Editor**

| Sl. no. | **Additional Editor’s comment** | **Authors’ response** |
| --- | --- | --- |
| 1. | - **To ensure your figures meet our technical requirements, please review our figure guidelines:** [**https://journals.plos.org/plosone/s/figures**](https://journals.plos.org/plosone/s/figures) | - As suggested, the figures of the manuscript have been prepared following the guidelines of PLOS figures. |
| 2. | - **You may also use PLOS’s free figure tool, NAAS, to help you prepare publication quality figures:** [**https://journals.plos.org/plosone/s/figures#loc-tools-for-figure-preparation**](https://journals.plos.org/plosone/s/figures#loc-tools-for-figure-preparation)**.** | - Many thanks for your suggestion. The figures in this manuscript have been prepared using PLOS’s free figure tool, NAAS, to ensure publication quality figures. |
