# Supplementary material for: Using the Social Ecological Model to identify barriers to accessing and using primary healthcare services by the rural Bangladeshi elderly: A systematic review
Source: PLoS One. 2026 Sep 18;21(9):e0358477. doi: 10.1371/journal.pone.0358477 (PMC13588402; doi:10.1371/journal.pone.0358477)
Supplement: Peer review history

# Author comment: Author response to Decision Letter 0

**DOI:** 10.1371/journal.pone.0358477.r001

18 Mar 2026

Attachment Submitted filename: Response to the editor.docx

# Peer review: Decision Letter 0

**DOI:** 10.1371/journal.pone.0358477.r002

**Authors:** Tadashi Kobayashi

1 Jun 2026

Dear Dr. Chanda,

Thank you for submitting your manuscript to PLOS ONE. After careful consideration, we feel that it has merit but does not fully meet PLOS ONE’s publication criteria as it currently stands. Therefore, we invite you to submit a revised version of the manuscript that addresses the points raised during the review process.

Please submit your revised manuscript by Jul 16 2026 11:59PM. If you will need more time than this to complete your revisions, please reply to this message or contact the journal office at plosone@plos.org. When you're ready to submit your revision, log on to https://www.editorialmanager.com/pone/ and select the 'Submissions Needing Revision' folder to locate your manuscript file.

- A letter that responds to each point raised by the academic editor and reviewer(s). You should upload this letter as a separate file labeled 'Response to Reviewers'.
- A marked-up copy of your manuscript that highlights changes made to the original version. You should upload this as a separate file labeled 'Revised Manuscript with Track Changes'.
- An unmarked version of your revised paper without tracked changes. You should upload this as a separate file labeled 'Manuscript'.

If you would like to make changes to your financial disclosure, please include your updated statement in your cover letter. Guidelines for resubmitting your figure files are available below the reviewer comments at the end of this letter.

If applicable, we recommend that you deposit your laboratory protocols in protocols.io to enhance the reproducibility of your results. Protocols.io assigns your protocol its own identifier (DOI) so that it can be cited independently in the future. For instructions see: https://journals.plos.org/plosone/s/submission-guidelines#loc-laboratory-protocols. Additionally, PLOS ONE offers an option for publishing peer-reviewed Lab Protocol articles, which describe protocols hosted on protocols.io. Read more information on sharing protocols at https://plos.org/protocols?utm_medium=editorial-email&utm_source=authorletters&utm_campaign=protocols.

As the corresponding author, your ORCID iD is verified in the submission system and will appear in the published article. PLOS supports the use of ORCID, and we encourage all coauthors to register for an ORCID iD and use it as well. Please encourage your coauthors to verify their ORCID iD within the submission system before final acceptance, as unverified ORCID iDs will not appear in the published article. *Only*  the individual author can complete the verification step; PLOS staff *cannot*  verify ORCID iDs on behalf of authors.

We look forward to receiving your revised manuscript.

Kind regards,

Tadashi Kobayashi, M.D., Ph.D

Academic Editor

PLOS One

Journal Requirements:

When submitting your revision, we need you to address these additional requirements.

1. Please ensure that your manuscript meets PLOS ONE's style requirements, including those for file naming. The PLOS ONE style templates can be found at

https://journals.plos.org/plosone/s/file?id=wjVg/PLOSOne_formatting_sample_main_body.pdf and

https://journals.plos.org/plosone/s/file?id=ba62/PLOSOne_formatting_sample_title_authors_affiliations.pdf.

2. If the reviewer comments include a recommendation to cite specific previously published works, please review and evaluate these publications to determine whether they are relevant and should be cited. There is no requirement to cite these works unless the editor has indicated otherwise.

Additional Editor Comments (if provided):

Thank you for submitting your manuscript, "Using the social ecological model to identify barriers to accessing and using primary healthcare services by the rural Bangladeshi elderly: A systematic review" (PONE-D-26-12917) to PLOS ONE.

Your manuscript has been evaluated by four independent experts in the field. While the reviewers appreciate the clinical and social relevance of investigating healthcare barriers for rural elderly populations in Bangladesh, they have raised several substantive methodological, theoretical, and reporting limitations that must be rigorously addressed before this manuscript can be considered for publication.

Based on the consensus of the reviewers and my own editorial evaluation, I am inviting you to submit a Major Revision of your manuscript.

In your revision, please pay particular attention to the following key critical issues:

1. Screening Rigor and Study Inclusion/Exclusion (Critical)

Inclusion of Study 10: Reviewer 1 rightly points out that Study 10 (Hamiduzzaman et al., 2022a) includes zero elderly participants (only healthcare professionals). Since your review specifically targets barriers faced by the rural elderly themselves, the inclusion of this study represents a serious screening error. You must re-evaluate your screening process, exclude this study (and any others failing the age eligibility criteria), and update your PRISMA flowchart, analysis, and synthesis accordingly.

2. Overlapping Sample Cohorts and Independence of Evidence

A significant proportion (45%) of your included studies originate from only two author groups (Hamiduzzaman; Hossen & Westhues), focused on very similar samples in the Sylhet district. Treating these as entirely independent sources of evidence inflates the weight of your qualitative synthesis. You must explicitly address this limitation, discuss the potential double-counting/overlap of study cohorts, and temper your conclusions.

3. Theoretical Framework and Conceptual Accuracy

Origins of the SEM: Correct the attribution of the Social Ecological Model. Lines 111-112 attribute the model to Bronfenbrenner (1977), which is actually the Ecological Systems Theory from developmental psychology. The public health-specific SEM used in your study was popularized by McLeroy et al. (1988).

Andersen’s Behavioral Model: Your critique of Andersen’s model focuses solely on its original 1968 formulation. However, Andersen revised this framework in 1995 (and subsequently) to explicitly incorporate contextual, cultural, and environmental factors. Please update this conceptual critique to reflect contemporary versions of the model.

4. Quality Appraisal Methodological Flaws

Appraising quality using arbitrary percentages (e.g., scoring CASP and MMAT out of 100%) is highly discouraged by Cochrane and the respective tool developers, as it masks critical domain-specific flaws. Please revise your quality appraisal to present a domain-based risk-of-bias/appraisal assessment.

Address the appraisal logical inconsistencies (e.g., rating a study with a sample size marked as "Not reported" as having "High quality" and full marks for recruitment and data collection).

5. Over-generalization and Gender Representation

Half of your included studies (10 out of 20) focus exclusively on elderly women. However, your discussion, conclusion, and policy recommendations are framed as applying universally to all rural Bangladeshi elderly (male and female). Please contextualize your findings to acknowledge this strong female bias and restrict over-generalizations.

6. Transparency and Reproducibility of Search Strategy

Please provide the full database-specific search strings used for each database to ensure reproducibility, as highlighted by Reviewer 4.

Verify and correct your PROSPERO registration ID (CRD420251164335A). Trailing letters in PROSPERO IDs are highly unusual and likely represent a transcription error.

Resolve the arithmetic and layout inconsistencies in your PRISMA flowchart (e.g., overlapping/concatenated numbers in the tracked changes version).

[Note: HTML markup is below. Please do not edit.]

Reviewers' comments:

Reviewer's Responses to Questions

**Comments to the Author**

1. Is the manuscript technically sound, and do the data support the conclusions?

Reviewer #1: Partly

Reviewer #2: Partly

Reviewer #3: Yes

Reviewer #4: Yes

**********

2. Has the statistical analysis been performed appropriately and rigorously?

Reviewer #1: No

Reviewer #2: No

Reviewer #3: N/A

Reviewer #4: No

**********

3. Have the authors made all data underlying the findings in their manuscript fully available?

The PLOS Data policy

Reviewer #1: No

Reviewer #2: Yes

Reviewer #3: Yes

Reviewer #4: Yes

**********

4. Is the manuscript presented in an intelligible fashion and written in standard English?

Reviewer #1: No

Reviewer #2: Yes

Reviewer #3: No

Reviewer #4: Yes

**********

Reviewer #1: 1. PRISMA numbers don't match across documents.

2. The SEM is credited to the wrong person:

Lines 111–112 say "The SEM developed by Bronfenbrenner (1977)." Bronfenbrenner developed Ecological Systems Theory — a child development model from psychology. The Social Ecological Model used in public health was developed by McLeroy et al. (1988). These are different frameworks from different fields. The SEM is the entire basis of this review. Getting its origin wrong is a foundational error.

3. One included study has zero elderly participants:

The eligibility criteria (Table 1) require elderly participants. Study 10 (Hamiduzzaman et al. 2022a) includes only doctors (n=3), pharmacists (n=3), a health inspector (n=1), and health assistants (n=4). No elderly people. It should have been excluded at screening. It was instead rated 100% quality and included in the synthesis.

4. Publication year range is wrong:

Lines 273–275 say studies were published "between 2005 and 2026." The earliest study in Table 4 is from 2006 (Biswas et al.). No 2005 study exists. Also, the eligibility window starts at 2000, but nothing from 2000–2005 was found — and this six-year gap is never mentioned.

5. A sentence meant to justify the study design contradicts itself :

Lines 132–134 read: "a systematic review was deemed the most suitable approach...rather than merely summarizing findings in relation to a specific research question, as would be done in a systematic review." It justifies systematic review by contrasting it with systematic review. The word at the end should be "narrative review." This survived revision uncorrected.

6. 45% of studies come from two author groups, with likely overlapping samples:

Hamiduzzaman authored 6 of 20 studies; Hossen & Westhues authored 3. Together: 9 of 20 (45%). Several Hamiduzzaman studies involve elderly women in Sylhet district with ~25 participants and similar methods — likely from the same or overlapping samples. Synthesizing these as independent evidence inflates the strength of findings. This is not acknowledged anywhere in the paper.

7. Every single study rated "High" quality:

All 20 studies scored High. All 7 quantitative studies received exactly 10/12 with identical Yes/No patterns — suggesting a template was applied, not genuine individual appraisal. Two studies with no ethical approval statement were still rated High. The quality classification method itself cites a paper by the corresponding author of this review (Reference 40, Chanda et al. 2022) — an unacknowledged self-citation as methodological authority.

8. One study has no reported sample size but gets a perfect quality score:

Study 12 (Table 4) lists sample size as "N= Not reported." Table 3 gives it 10/10 (100%) on the CASP qualitative checklist, including full marks for recruitment appropriateness and data collection adequacy. You cannot assess these without knowing how many participants there were.

9. Most studies are about elderly women, but conclusions claim to cover all elderly:

At least 10 of 20 included studies focus exclusively on elderly women. The conclusions and all five policy recommendations (lines 598–620) are written as if they apply to all rural elderly in Bangladesh. Claims comparing elderly men and women (lines 306–307) cannot be made from a literature base that barely includes elderly men.

10. Two abbreviations listed are never used in the text :

"IGA" (Income Generating Activities) and "CEBM" appear in the abbreviations list (lines 641–653) but neither is used in the manuscript body. The list was not checked against the actual text.

11. PROSPERO ID has a non-standard trailing letter:

The registration number given is "CRD420251164335A." PROSPERO IDs never end with a letter. This may be a transcription error. Since the registration number must be publicly verifiable, it must be corrected to exactly what appears in the PROSPERO database.

12. The critique of Andersen's model targets its 1968 version:

Lines 106–108 say Andersen's model ignores culture and social interaction. This was true of the 1968 original. Andersen revised the model in 1995 and again subsequently to explicitly include contextual factors, social environment, and cultural norms. A 2026 paper should engage with the current version of a competing framework, not a 58-year-old one.

13. A finding with no supporting literature is presented without any caveat:

Lines 527–530 explicitly state: "no study was found focused on the rural elderly in line with the findings" — referring to gender preference for healthcare providers. Despite this admission, the finding is discussed at length and included in the conclusions (line 631) as if it has the same evidentiary weight as findings supported by multiple studies. It should be clearly labelled as preliminary and requiring further investigation.

Reviewer #2: Firstly, I would like to congratulate the authors for studying this topic and highlighting societal gaps.

Few comments from my end:

Given that the authors derive policy recommendations from primary data subjected to statistical analysis and meta-analytic synthesis, one might anticipate methodological rigor. However, the analytical framework exhibits critical limitations that undermine the validity and generalizability of both findings and prescriptive outcomes. Specifically, the statistical models appear to suffer from inadequate adjustment for confounding variables, potential selection bias in data aggregation, and insufficient sensitivity analyses to validate meta-analytic effect sizes. Consequently, the resultant policy recommendations lack the robustness required for evidence-based decision-making, rendering them vulnerable to overgeneralization and reduced translational impact in real-world policy contexts.

So I would strongly recommend to include meta-analysis in the study.

Reviewer #3: General comments - This manuscript titled "Using the social ecological model to identify barriers to accessing and using primary healthcare services by the rural Bangladeshi elderly: A systematic review" synthesizes the multi level barriers elderly people in rural Bangladeshi face when accessing PHC services.

Major comment - The manuscript has a number of grammatical errors especially in the study design section. While it is clear there is a need for this study, the authors did not do a thorough job in providing evidence for the significance of the study in the background section. Finally, there are multiple communities and villages in rural Bangladeshi but the authors did not specify which communities were included in the review.

Recommendation - The authors need to proof read the manuscript again for grammatical errors, specifically pages 6, 11 and 12. Also consider re-writing the background of the study to include more data that is specific to the research question.

Reviewer #4: The manuscript examines barriers to accessing and using primary healthcare (PHC) services among the rural elderly in Bangladesh using the Social Ecological Model (SEM). The topic is relevant to ageing populations in low- and middle-income countries and aligns with SDG3 priorities. The use of SEM is appropriate for organizing multilevel determinants.

However, the manuscript has substantive methodological and reporting limitations that affect transparency, reproducibility, and analytical depth. Key issues include inconsistent study design description, non-reproducible search strategy, inappropriate use of quality appraisal tools, and largely descriptive synthesis without sufficient interpretive advancement.

Find my specific comments next

Major Comments

Study Design and Conceptual Clarity

The manuscript presents an internal inconsistency in defining the study design.

Action: Please clearly define the study as a systematic review with thematic synthesis and ensure consistency across all sections.

Search Strategy and Reproducibility

The search strategy is not reproducible.

What to do:

• Provide full database-specific search strings

• Specify search dates, language limits, and grey literature strategy

• Define Google Scholar screening limits

Application of the PICO Framework

PICO is incorrectly operationalised.

Authors may reframe PICO with Population (elderly), Exposure (barriers), Outcome (PHC access and utilization).

Eligibility Criteria

PHC not clearly defined and timeframe unjustified.

Kindly Define PHC and justify the time restriction.

Study Selection Process

There is no inter-rater reliability reported.

The authors should report agreement statistics or describe the consensus process(Cohen’s kappa).

Quality Appraisal

There is an inappropriate scoring of CASP and MMAT.

Authors may remove the scoring and present the domain-based risk-of-bias assessment.

Data Synthesis

Why only Descriptive and deductive?

Clarify coding approach, strengthen analytical synthesis and compare findings across studies, and avoid isolated statistics.

Conceptual Framing

There is a mischaracterisation of Andersen’s model.

Authors should correct and provide balanced justification for SEM.

Discussion

The interpretation is limited under the discussion section.

Expand theoretical and policy implications.

Limitations

Include bias and methodological limitations.

Minor Comments

• Standardise terminology (elderly vs older adults)

• Reduce repetition

• orrect grammatical inconsistencies

• Remove duplicate abbreviations (e.g., HPs)

Reporting Standards

Align manuscript with PRISMA:

• Search for reproducibility

• Risk of bias reporting

• Transparent synthesis

**********

what does this mean?). If published, this will include your full peer review and any attached files.

If you choose “no”, your identity will remain anonymous but your review may still be made public.

**Do you want your identity to be public for this peer review?**  For information about this choice, including consent withdrawal, please see our Privacy Policy

Reviewer #1: No

Reviewer #2: **Yes:** Anamika

Reviewer #3: No

Reviewer #4: No

**********

[NOTE: If reviewer comments were submitted as an attachment file, they will be attached to this email and accessible via the submission site. Please log into your account, locate the manuscript record, and check for the action link "View Attachments". If this link does not appear, there are no attachment files.]

To ensure your figures meet our technical requirements, please review our figure guidelines: https://journals.plos.org/plosone/s/figures

You may also use PLOS’s free figure tool, NAAS, to help you prepare publication quality figures: https://journals.plos.org/plosone/s/figures#loc-tools-for-figure-preparation.

NAAS will assess whether your figures meet our technical requirements by comparing each figure against our figure specifications.

# Author comment: Author response to Decision Letter 1

**DOI:** 10.1371/journal.pone.0358477.r003

20 Aug 2026

Please see attached files.

Attachment Submitted filename: Response to reviewers_v1_14-8-2026.docx

# Editor report: Decision Letter 1

**DOI:** 10.1371/journal.pone.0358477.r004

**Authors:** Tadashi Kobayashi

25 Aug 2026

Dear Dr. Chanda,

Thank you for submitting your manuscript to PLOS One. After careful consideration, we feel that it has merit but does not fully meet PLOS One’s publication criteria as it currently stands. Therefore, we invite you to submit a revised version of the manuscript that addresses the points raised during the review process.

==============================

I have carefully evaluated your revised manuscript along with the detailed point-by-point responses. I commend the authors for their thorough, rigorous, and constructive revisions, which have successfully resolved all major theoretical, methodological, and screening concerns raised during the initial review (including the proper exclusion of Study 10, the correction of the SEM framework attribution, and the implementation of a domain-based risk-of-bias assessment).

<ms-cmark-node _ngcontent-ng-c183360516=" " _nghost-ng-c183360516=" " style="-webkit-tap-highlight-color: transparent; box-sizing: border-box; display: contents;">The manuscript is now very close to publication. Before final acceptance, please address the following minor editorial and typographical points:</ms-cmark-node>

1. <ms-cmark-node _ngcontent-ng-c183360516=" " _nghost-ng-c183360516=" " style="-webkit-tap-highlight-color: transparent; box-sizing: border-box; display: contents;">
2. <ms-cmark-node _ngcontent-ng-c183360516=" " _nghost-ng-c183360516=" " style="-webkit-tap-highlight-color: transparent; box-sizing: border-box; display: contents;">
  <ms-cmark-node _ngcontent-ng-c183360516=" " _nghost-ng-c183360516=" " style="-webkit-tap-highlight-color: transparent; box-sizing: border-box; display: contents;">**Subtheme Numbering (Theme 3):**  On page 36 (lines 402–404), the subthemes are currently numbered as "(i), (iii), (iii), (iv)". Please correct this sequence to "(i), (ii), (iii), (iv)".</ms-cmark-node>
  </ms-cmark-node>
3. <ms-cmark-node _ngcontent-ng-c183360516=" " _nghost-ng-c183360516=" " style="-webkit-tap-highlight-color: transparent; box-sizing: border-box; display: contents;">
  <ms-cmark-node _ngcontent-ng-c183360516=" " _nghost-ng-c183360516=" " style="-webkit-tap-highlight-color: transparent; box-sizing: border-box; display: contents;">**Figure 2 Labeling:**  In the uploaded image file for Figure 2 (the PRISMA flowchart), the internal caption embedded within the image reads "Figure 1 PRISMA flowchart...". Please correct this inside the graphic to "Figure 2".</ms-cmark-node>
  </ms-cmark-node>
4. <ms-cmark-node _ngcontent-ng-c183360516=" " _nghost-ng-c183360516=" " style="-webkit-tap-highlight-color: transparent; box-sizing: border-box; display: contents;">
  <ms-cmark-node _ngcontent-ng-c183360516=" " _nghost-ng-c183360516=" " style="-webkit-tap-highlight-color: transparent; box-sizing: border-box; display: contents;">**Typographical Errors in Table 4:** </ms-cmark-node>
5. <ms-cmark-node _ngcontent-ng-c183360516=" " _nghost-ng-c183360516=" " style="-webkit-tap-highlight-color: transparent; box-sizing: border-box; display: contents;"><ms-cmark-node _ngcontent-ng-c183360516=" " _nghost-ng-c183360516=" " style="-webkit-tap-highlight-color: transparent; box-sizing: border-box; display: contents;">
  <ms-cmark-node _ngcontent-ng-c183360516=" " _nghost-ng-c183360516=" " style="-webkit-tap-highlight-color: transparent; box-sizing: border-box; display: contents;">Row 1 (Sarker, 2025): Please correct "Corress-sectional survey" to "Cross-sectional survey".</ms-cmark-node>
  </ms-cmark-node>
6. <ms-cmark-node _ngcontent-ng-c183360516=" " _nghost-ng-c183360516=" " style="-webkit-tap-highlight-color: transparent; box-sizing: border-box; display: contents;">
  <ms-cmark-node _ngcontent-ng-c183360516=" " _nghost-ng-c183360516=" " style="-webkit-tap-highlight-color: transparent; box-sizing: border-box; display: contents;">Row 16 (Biswas et al., 2007): Please correct "Nnon-random strategy" to "Non-random strategy".</ms-cmark-node>
  </ms-cmark-node>
7. </ms-cmark-node>
8. </ms-cmark-node>
9. <ms-cmark-node _ngcontent-ng-c183360516=" " _nghost-ng-c183360516=" " style="-webkit-tap-highlight-color: transparent; box-sizing: border-box; display: contents;">
  <ms-cmark-node _ngcontent-ng-c183360516=" " _nghost-ng-c183360516=" " style="-webkit-tap-highlight-color: transparent; box-sizing: border-box; display: contents;">**Minor Spacing:**  On page 26, line 292, please add a space between the citation bracket and the following word ("[48] was followed").</ms-cmark-node>
  </ms-cmark-node>
10. </ms-cmark-node>

==============================

Please submit your revised manuscript by Oct 09 2026 11:59PM. If you will need more time than this to complete your revisions, please reply to this message or contact the journal office at plosone@plos.org. When you're ready to submit your revision, log on to https://www.editorialmanager.com/pone/ and select the 'Submissions Needing Revision' folder to locate your manuscript file.

- A letter that responds to each point raised by the academic editor and reviewer(s). You should upload this letter as a separate file labeled 'Response to Reviewers'.
- A marked-up copy of your manuscript that highlights changes made to the original version. You should upload this as a separate file labeled 'Revised Manuscript with Track Changes'.
- An unmarked version of your revised paper without tracked changes. You should upload this as a separate file labeled 'Manuscript'.

If you would like to make changes to your financial disclosure, please include your updated statement in your cover letter. Guidelines for resubmitting your figure files are available below the reviewer comments at the end of this letter.

If applicable, we recommend that you deposit your laboratory protocols in protocols.io to enhance the reproducibility of your results. Protocols.io assigns your protocol its own identifier (DOI) so that it can be cited independently in the future. For instructions see: https://journals.plos.org/plosone/s/submission-guidelines#loc-laboratory-protocols. Additionally, PLOS One offers an option for publishing peer-reviewed Lab Protocol articles, which describe protocols hosted on protocols.io. Read more information on sharing protocols at https://plos.org/protocols?utm_medium=editorial-email&utm_source=authorletters&utm_campaign=protocols.

As the corresponding author, your ORCID iD is verified in the submission system and will appear in the published article. PLOS supports the use of ORCID, and we encourage all coauthors to register for an ORCID iD and use it as well. Please encourage your coauthors to verify their ORCID iD within the submission system before final acceptance, as unverified ORCID iDs will not appear in the published article. *Only*  the individual author can complete the verification step; PLOS staff *cannot*  verify ORCID iDs on behalf of authors.

We look forward to receiving your revised manuscript.

Kind regards,

Tadashi Kobayashi, M.D., Ph.D

Academic Editor

PLOS One

Journal Requirements:

If the reviewer comments include a recommendation to cite specific previously published works, please review and evaluate these publications to determine whether they are relevant and should be cited. There is no requirement to cite these works unless the editor has indicated otherwise.

Please review your reference list to ensure that it is complete and correct. If you have cited papers that have been retracted, please include the rationale for doing so in the manuscript text, or remove these references and replace them with relevant current references. Any changes to the reference list should be mentioned in the rebuttal letter that accompanies your revised manuscript. If you need to cite a retracted article, indicate the article’s retracted status in the References list and also include a citation and full reference for the retraction notice.

Additional Editor Comments:

[Note: HTML markup is below. Please do not edit.]

[NOTE: If reviewer comments were submitted as an attachment file, they will be attached to this email and accessible via the submission site. Please log into your account, locate the manuscript record, and check for the action link "View Attachments". If this link does not appear, there are no attachment files.]

To ensure your figures meet our technical requirements, please review our figure guidelines: https://journals.plos.org/plosone/s/figures

You may also use PLOS’s free figure tool, NAAS, to help you prepare publication quality figures: https://journals.plos.org/plosone/s/figures#loc-tools-for-figure-preparation.

NAAS will assess whether your figures meet our technical requirements by comparing each figure against our figure specifications.

# Author comment: Author response to Decision Letter 2

**DOI:** 10.1371/journal.pone.0358477.r005

26 Aug 2026

Further revisions to the manuscript have been made based on your minor comments and submitted the files. Please see the files attached.

Attachment Submitted filename: Response to Reviewers.docx

# Editor report: Decision Letter 2

**DOI:** 10.1371/journal.pone.0358477.r006

**Authors:** Tadashi Kobayashi

1 Sep 2026

Using the social ecological model to identify barriers to accessing and using primary healthcare services by the rural Bangladeshi elderly: A systematic review

PONE-D-26-12917R2

Dear Dr. Chanda,

We’re pleased to inform you that your manuscript has been judged scientifically suitable for publication and will be formally accepted for publication once it meets all outstanding technical requirements.

Within one week, you’ll receive an e-mail detailing the required amendments. When these have been addressed, you’ll receive a formal acceptance letter and your manuscript will be scheduled for publication.

An invoice will be generated when your article is formally accepted. Please note, if your institution has a publishing partnership with PLOS and your article meets the relevant criteria, all or part of your publication costs will be covered. Please make sure your user information is up-to-date by logging into Editorial Manager at Editorial Manager® and clicking the ‘Update My Information' link at the top of the page. For questions related to billing, please contact billing support.

If your institution or institutions have a press office, please notify them about your upcoming paper to help maximize its impact. If they’ll be preparing press materials, please inform our press team as soon as possible -- no later than 48 hours after receiving the formal acceptance. Your manuscript will remain under strict press embargo until 2 pm Eastern Time on the date of publication. For more information, please contact onepress@plos.org.

Kind regards,

Tadashi Kobayashi, M.D., Ph.D

Academic Editor

PLOS One

Additional Editor Comments (optional):

All of the remaining minor editorial and typographical points have been satisfactorily addressed. The manuscript is methodologically sound, strictly adheres to PRISMA reporting standards, and provides a valuable synthesis of primary healthcare access barriers for the rural elderly using the Social Ecological Model.

Thank you for your constructive engagement throughout the peer review and revision process. Your detailed and thoughtful responses have significantly enhanced the clarity, rigor, and impact of the manuscript.

Reviewers' comments:

# Editor report: Acceptance letter

**DOI:** 10.1371/journal.pone.0358477.r007

**Authors:** Tadashi Kobayashi

PONE-D-26-12917R2

PLOS One

Dear Dr. Chanda,

I'm pleased to inform you that your manuscript has been deemed suitable for publication in PLOS One. Congratulations! Your manuscript is now being handed over to our production team.

At this stage, our production department will prepare your paper for publication. This includes ensuring the following:

* All references, tables, and figures are properly cited

* All relevant supporting information is included in the manuscript submission,

* There are no issues that prevent the paper from being properly typeset

You will receive further instructions from the production team, including instructions on how to review your proof when it is ready. Please keep in mind that we are working through a large volume of accepted articles, so please give us a few days to review your paper and let you know the next and final steps.

Lastly, if your institution or institutions have a press office, please let them know about your upcoming paper now to help maximize its impact. If they'll be preparing press materials, please inform our press team within the next 48 hours. Your manuscript will remain under strict press embargo until 2 pm Eastern Time on the date of publication. For more information, please contact onepress@plos.org.

You will receive an invoice from PLOS for your publication fee after your manuscript has reached the completed accept phase. If you receive an email requesting payment before acceptance or for any other service, this may be a phishing scheme. Learn how to identify phishing emails and protect your accounts at https://explore.plos.org/phishing.

If we can help with anything else, please email us at customercare@plos.org.

Thank you for submitting your work to PLOS One and supporting open access.

Kind regards,

PLOS One Editorial Office Staff

on behalf of

Dr. Tadashi Kobayashi

Academic Editor

PLOS One
